# Water Accessibility Refinement of the Extended Structure of KirBac1.1 in the Closed State

**DOI:** 10.3389/fmolb.2021.772855

**Published:** 2021-11-30

**Authors:** Reza Amani, Charles D. Schwieters, Collin G. Borcik, Isaac R. Eason, Ruixian Han, Benjamin D. Harding, Benjamin J. Wylie

**Affiliations:** ^1^ Texas Tech University, Department of Chemistry and Biochemistry, Lubbock, TX, United States; ^2^ Computational Biomolecular Magnetic Resonance Core, National Institutes of Digestive Diseases and Kidneys, NIH, Bethesda, MD, United States; ^3^ University of Wisconsin-Madison, Department of Biochemistry and Chemistry, Madison, WI, United States; ^4^ Biophysics Program, University of Wisconsin at Madison, Madison, WI, United States

**Keywords:** solid state NMR, membrane protein, xplor-NIH, water-edited spectroscopy, structure refinement, potassium channel

## Abstract

NMR structures of membrane proteins are often hampered by poor chemical shift dispersion and internal dynamics which limit resolved distance restraints. However, the ordering and topology of these systems can be defined with site-specific water or lipid proximity. Membrane protein water accessibility surface area is often investigated as a topological function *via* solid-state NMR. Here we leverage water-edited solid-state NMR measurements in simulated annealing calculations to refine a membrane protein structure. This is demonstrated on the inward rectifier K^+^ channel KirBac1.1 found in *Burkholderia pseudomallei*. KirBac1.1 is homologous to human Kir channels, sharing a nearly identical fold. Like many existing Kir channel crystal structures, the 1p7b crystal structure is incomplete, missing 85 out of 333 residues, including the N-terminus and C-terminus. We measure solid-state NMR water proximity information and use this for refinement of KirBac1.1 using the Xplor-NIH structure determination program. Along with predicted dihedral angles and sparse intra- and inter-subunit distances, we refined the residues 1–300 to atomic resolution. All structural quality metrics indicate these restraints are a powerful way forward to solve high quality structures of membrane proteins using NMR.

## Introduction

Solid-state NMR (SSNMR) is essential to the structural and functional characterization of membrane proteins (MPs) (Schubeis et al., 2018; Radoicic et al., 2014; Wylie et al., 2016; Mandala et al., 2018; Tran et al., 2020). SSNMR can study MPs in native or native-like environments, allowing site-specific analysis of protein structure and activity. SSNMR is not inherently limited by the size of system, an issue for liquid-state NMR. SSNMR can thus access proteins in proteoliposomes and cellular envelopes (Renault et al., 2012). SSNMR does not require high salt concentrations, long-range order, or cryogenic temperatures, all required for X-ray crystallography. Over the past two decades the water accessible surface of MPs was actively quantified *via* SSNMR (Kumashiro et al., 1998; Ader et al., 2009; Li et al., 2010; Su et al., 2011; Hornig et al., 2013). Over this time, water-edited SSNMR examined the rearrangement of membrane proteins, molecular motion in deuterated samples, and determined membrane insertion topology (Najbauer et al., 2019). In pursuit of functional states of K^+^ channels, Ader *et al.* used water-edited SSNMR spectroscopy to unambiguously uncover a dramatic increase in water-accessible surface area between the closed/inactivated state and open/activated states of the KcsA-Kv1.3 chimera. Subsequently, Borcik *et al.* discovered site-specifically that water accessibly is diminished upon activation of KirBac1.1. This work proposed a key component of the KirBac1.1 gating mechanism, where C-terminal domains rotate and form electrostatic contacts to stabilize the activated state. Thus, relative solvent accessibility during the K^+^ channel gating cycle may not be universal. Despite the utility and wide usage of water-edited SSNMR spectroscopy, site-specific solvent accessibility has never been utilized to solve or refine the structure of an MP. Here, we demonstrate the applicability of water-edited SSNMR spectroscopy as an experimental restraint to refine the structure of a MP within Xplor-NIH (Schwieters et al., 2018) simulated annealing calculations.

KirBac1.1 is a 149.02 kDa homotetrameric membrane protein native to *Burkholderia pseudomallei.* Like all inward-rectifier K^+^ (Kir) channels, it favors inward potassium ion conductance through the membrane, helping to set the resting membrane potential (Kuo, 2003; Cheng et al., 2009; Wang et al., 2009; Linder et al., 2015). KirBac1.1 retains the characteristic TVGYG selectivity filter motif found in most K^+^ channels. This facilitates K^+^ conduction near the rate of free diffusion and is impermeable to Na^+^ and smaller cations. Each KirBac1.1 monomer consists of two transmembrane (TM) helices, a slide helix, selectivity filter loop, pore helix, and a gating bundle. KirBac1.1 is activated by the association of anionic lipids to a large cationic binding pocket rich in arginine residues (Enkvetchakul et al., 2007; Clarke et al., 2010a; Wang S. et al., 2012; Borcik et al., 2020; van Aalst et al., 2020). Many regions of the protein are intimately tied to channel function and activity, including transmembrane helix 1 (TM1), transmembrane helix 2 (TM2), the slide helix, and the C-terminal gating bundle (Kuo et al., 2003; Enkvetchakul et al., 2004; Enkvetchakul et al., 2007; Paynter et al., 2010; Amani et al., 2020; Borcik et al., 2020). However, to uncover the complete structure-activity relationship of the gating cycle requires a more complete full-length structure. Unfortunately, the existing crystal structures 1p7b lacks the N-terminus (residues 1:35), several turns and coils in the gating bundle (residues 196:205, 290:295) and the C-terminus (residues 310:333) and the crystal structure 2wll lacks 5:37, 200:205, 290:295, 310:333 (Kuo, 2003; Clarke et al., 2010b). Thus, a full-length structure could provide needed information, including pivotal inter-subunit contacts between N-termini and the adjacent cytoplasmic subunit. In addition, it is known the orientations of these regions may change with lipid environment and may be sensitive to salt concentration as they are highly electrostatic. In our previous studies, we characterized the inactivated (closed) and activated (open) states of KirBac1.1 in great detail. We assigned the chemical shifts for both states in activating and inactivating bilayers (Amani et al., 2020) and identified domain motions correlating to both states (Borcik et al., 2020). We found that the water accessible surface of the Kir domain of the closed state was significantly greater than the activated state. These studies motivated this work, as we seek to leverage our acquired knowledge to probe distinct states of the channel structurally. The closed ground state of the channel is the logical starting point in the structural mapping of this Kir channel. It has a greater overall water accessible surface and is the starting point of the gating and thermodynamic cycle of the channel. Thus, full-length structures of KirBac1.1, and many other MPs, will benefit from SSNMR analysis and structure elucidation that recognize their unique topologies.

We refined the structure of KirBac1.1 from residues 1 to 301 using the following workflow: We first modelled in all missing regions of the 1p7b crystal structure using a ROSETTA remodel “quick and dirty” protocol (Huang et al., 2011). Our previously reported ^15^N and ^13^C chemical shift assignments for residues 1 to 301 (Amani et al., 2020; Borcik et al., 2020) for the closed-state of U-^15^N,^13^C-KirBac1.1 reconstituted into zwitterionic 1-palmitoyl-2-oleoyl-sn-glycero-3-phosphocholine (POPC) lipid bilayers were used to generate dihedral angles in TALOSN (Shen and Bax, 2013). We then acquired water-edited SSNMR spectra of U-^15^N,^13^C-KirBac1.1 in POPC proteoliposomes and extensively site-specifically assigned a spectrum with a short ^1^H_water_-^1^H_protein_ mixing time. To provide sparse distance restraints, we acquired a three-dimensional (3D) dipole-assisted rotational resonance (DARR) (Takegoshi et al., 2001) spectrum with 50 and 500 ms mixing times during the first and second mixing periods (Zhou et al., 2006). This spectrum yielded several key inter- and intra-subunit distances. We then utilized the TALOSN dihedral angles, sparse distances, and site-specific solvent accessibility measurements to refine the full-length model of KirBac1.1 within Xplor-NIH (Schwieters et al., 2018). Water-based paramagnetic resonance restraints had previously been used as solvent accessibility restraints. Here, Xplor-NIH’s PSolPot term was used to fit SSNMR-style solvent accessibility water-accessible surface area data of KirBac1.1 This work represents one of the largest protein structures ever refined using SSNMR solvent accessible surfaces as a restraint.

## Materials and Methods

### SSNMR Sample Preparation

U-^15^N,^13^C-labeled KirBac1.1 was expressed and purified as described previously (Amani et al., 2020; Borcik et al., 2020). Briefly, the protein was expressed from *E. coli* in M9 minimum media enriched with ^15^NH_4_Cl, ^13^C-glucose, and a 10 ml aliquot of 10X concentrated BioExpress (Cambridge Isotopes Laboratories, Tewksbury, MA 01876) (Bhate et al., 2013; Amani et al., 2020; Borcik et al., 2020). Protein overexpression was induced at an OD_600_ of 0.8 by adding isopropyl β-D-1-thiogalactopyronoside (IPTG) to a concentration of 1 mM. After 16 h of induction at 18°C, cells were harvested *via* centrifugation. Cells were lysed *via* homogenization at 10–15 kpsi. The protein was extracted by adding decyl-β-D-maltopyranoside (DM) to a 30 mM concentration and leaving the lysate on an orbital shaker for 4 h in the presence of Pierce^TM^ Protease inhibitors tablets, EDTA-Free (Thermo Scientific). After extraction, supernatant was spun in an ultracentrifuge, sterile filtered, and loaded onto a 5 ml HisTrap (GE Healthcare Life Sciences) column. The sample was subsequently passed through a HiPrep 26/10 desalting column (GE Healthcare Life Sciences), followed by a HiLoad 16/600 Superdex 200 size exclusion column (GE Healthcare Life Sciences). Purified protein was mixed with CHAPS solubilized POPC at a 1:1 ratio (w/w). The sample was then stepwise reconstituted *via* the slow addition of BioBeads-SM2 (Bio-Rad, Hercules, CA). BioBeads were then removed and the sample pelleted *via* centrifugation and packed into a 3.2 mm limited speed PENCIL rotor (Revolution NMR, Ft. Collins, CO).

### NMR Spectroscopy

All SSNMR spectra were acquired at field strengths of either 17.6 T (750 MHz ^1^H frequency) or 14.1 T (600 MHz ^1^H frequency) on SSNMR spectrometers located at National Magnetic Resonance Facility at Madison (NMRFAM, University of Wisconsin, Madison, WI). The CCC 3D DARR spectrum (Zhou et al., 2006) was acquired with 50 and 500 ms of DARR mixing in the first and second mixing periods, respectively, at 750 MHz with a Varian (Fort Collins, CO) 3.2 mm Balun probe in double resonance mode ^1^H-^13^C mode. Magic-angle spinning (MAS) (Andrew et al., 1958; Lowe, 1959) was performed at 12.5 kHz with a variable temperature (VT) set point of −30°C and a flow rate of 40 lpm (calibrated to −15 ± 3°C). This temperature was chosen because it provided the greatest overall signal for this 3D experiment. 83 kHz of SPINAL-64 (Fung et al., 2000) ^1^H decoupling was applied during all chemical shift evolution periods, and hard 90° pulses were 2.4 μs for ^1^H and 3.05 μs ^13^C. Polarization transfer was facilitated *via* adiabatic cross polarization (CP) (Pines et al., 1972) with a 1 ms contact time. During CP 1H power was set to 78 kHz and ^13^C power set to 65 kHz. The recycle delay was set to 1.5 s. The three-dimensional (3D) data was acquired with non-uniform sampling of the indirect dimensions, with a 256 × 256 grid of acquired points with 12.5% points acquired corresponding to 35.4% sampled points in each dimension.

The water accessibility experiments were performed at a magnetic field strength of 14.1 T (600 MHz ^1^H frequency). The rotor was placed in a 3.2 mm Varian (Fort Collins, CO) T3 HXY probe in double resonance mode, and spun at the magic angle at a spinning rate of 12.5 kHz. To ensure all water surrounding the protein was liquid, the VT was set to 10 C (sample temperature of 25 ± 3°C) for all water edited experiments with a flow rate of 40 lpm. The cross-peaks in these spectra were matched to similar 2D spectra acquired at −5°C and −15°C to confirm no major chemical shift differences. In our past work, KirBac1.1 was assigned over this temperature range to facilitate this process. Pulse widths of 2.7 and 2.55−μs were applied to 1H and 13C, respectively. A 1.5 s recycle delay was implemented for all water edited experiments. Water-edited spectra were acquired using an initial 1H T_2_ filter of 1.5 ms, to eliminate ^1^H polarization arising from protein and lipid signals. 1H to 13C transfer was mediated *via* cross polarization with spin lock fields of 65 and 84 kHz on ^1^H and ^13^C, respectively, for a contact duration of 1 ms. Additional parameters for the water edited spectra include a 50 ms DARR mixing followed by 70 kHz of ^1^H SPINAL-64 decoupling. We assessed the water accessibility with ^1^H-^1^H spin diffusion times of 4 and 16 ms.

### Structure Calculation *via* Xplor-NIH

Throughout all calculations, strict C4 symmetry was maintained using the symSimulation facility (Schwieters et al., 2018), and subunit backbone geometry of residues 36–301 was restrained to that of 1p7b using a non-crystallographic term allowing up to 1 Å of deviation with zero energy penalty. An additional NCS term was employed between the centroids of opposite subunits to prevent overall expansion. Energy terms employed during structure calculations included ^13^C-^13^C intra- and inter-subunit distances (NOE potential), TALOSN derived dihedral angles (CDIH) (Bermejo and Schwieters, 2018), the hydrogen bond potential of mean force (HBPot) (Schwieters et al., 2020), and either the EEFx (Tian et al., 2014; Tian et al., 2015) or EEFx with IMMx (Tian et al., 2017) terms which both model realistic non-bonded interactions within implicit solvent. In their current implementation the IMMx potential builds upon the EEFx potential by including terms explicitly defining the hydrophobic thickness of the bilayer and its dielectric properties. The bilayer dielectric is adjustable and can be scaled differently during initial structural solution and the final refinement. In each calculation, the backbone dihedral angles of residues 1–35 and 302–333 were randomized then relaxed into non-clashing conformations employing the repulsion-only RepelPot term (Schwieters et al., 2018) using gradient minimization, followed by 40 ps of high-temperature (3500 K) molecular dynamics. During this initial repulsion-only phase, EEFx and IMMx not enabled, as they are not stable in the presence of initial close-contacts. The nonbonded representation was then switched over to the implicit model and 30 ps of molecular dynamics was run. This was followed by annealing to 25 K using EEFx or EEFx with IMMx. Following initial calculations, refinement was performed including the PSolPot term (Wang Y. et al., 2012; Gong et al., 2018; Kooshapur et al., 2018) representing site-specific protein-water interactions along the other restraints in a procedure identical to that above with the exception that there is no torsion angle randomization. We performed three PSolPot calculations. In the first we only used completely unambiguously assigned solvent-accessible residues. This generated an ensemble of structures with improved overall structural resolution. These structures were then used to aid in assigning ambiguous water-proximal resonances. The complete set of water-accessible restraints were then used to refine the ensemble of structures. At the end we ran another simulated annealing structural refinement with several Ramachandran outliers deleted from the PSolPot table. The result of last set of PSolPot calculation showed small improvement in some cases. Structure quality was assessed by MolProbity (Williams et al., 2018). All RMSDs were measured *via* VMD-XPLOR (Schwieters and Clore, 2001).

## Results and Discussion

### SSNMR Data

NMR structures are solved by including distance measurements and other structural restraints as pseudopotentials into simulated annealing calculations. However, as proteins grow larger, spectral crowding will occur. This is compounded when the protein structure is dominated by a single type of secondary structure, as often occurs in α-helical membrane proteins. Thus, as more distances are measured more peaks appear leading to greater information at the cost of reduced site-specific resolution. However, following observations reported by several groups and within our own laboratory, we found that large domain motions may be mapped by the observable solvent accessible surface (Borcik et al., 2020).

**FIGURE 1:**
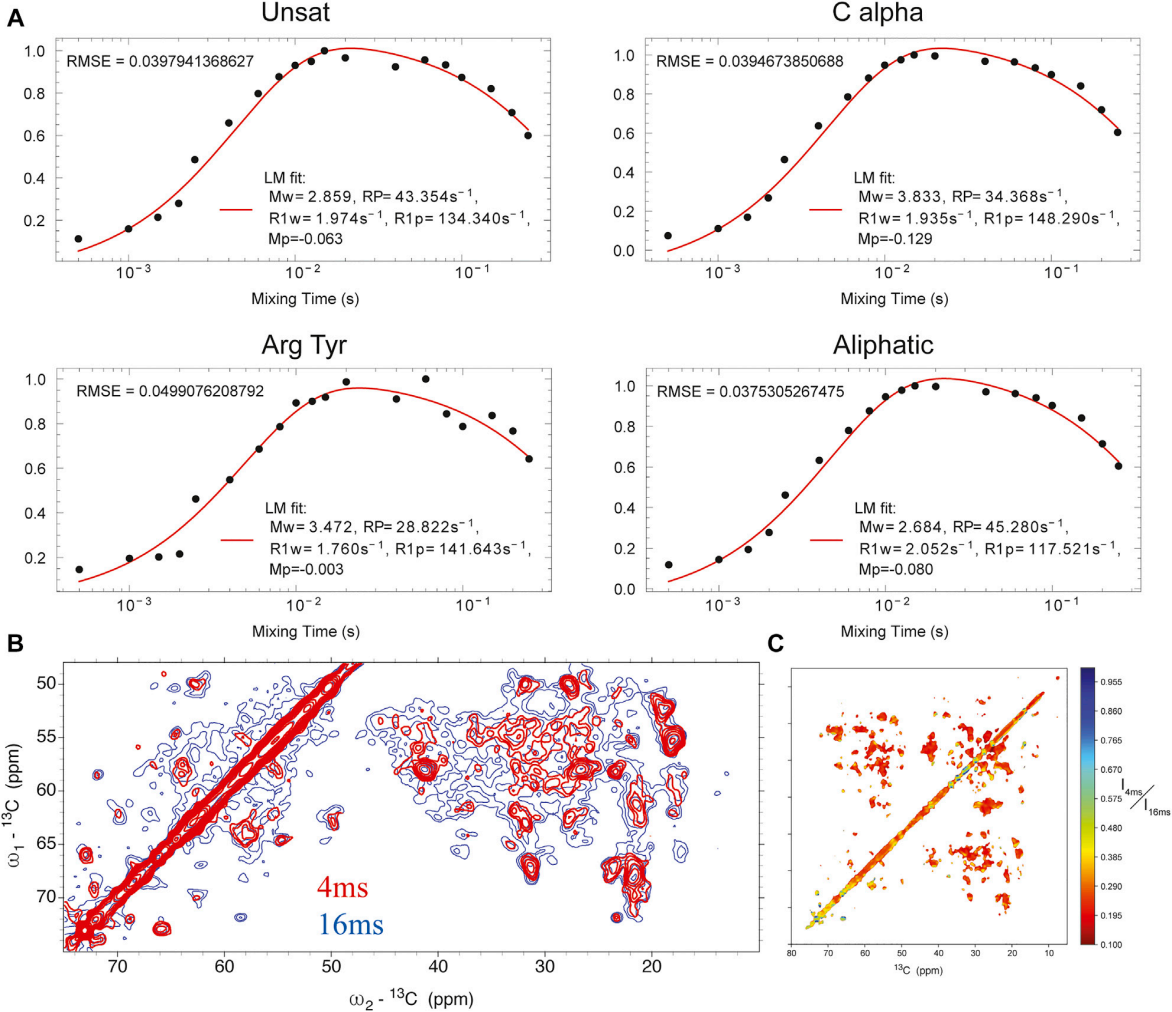
**(A)** buildup curves for different regions of the protein as a function of ^1^H_water_-^1^H_protein_ mixing time. **(B)** ^13^C-^13^C DARR spectrum with 4 ms ^1^H_water_-^1^H_protein_ mixing (red) overlaid onto a similar spectrum with 16 ms of ^1^H_water_-^1^H_protein_ mixing (blue). **(C)** heat map of individual point intensity in 4 ms ^1^H_water_-^1^H_protein_ spectrum compared to 16 ms spectrum.

***Water-edited SSNMR***. We measured the solvent-accessible surface of the closed state of the I131C mutant of KirBac1.1 in POPC bilayers using water-edited SSNMR. These water-edited spectra of U-^15^N,^13^C-KirBac1.1 are similar to spectra described previously, but they probe the native closed state rather than the closed state of the R49/151/153/Q mutant (Borcik et al., 2020). This technique capitalizes on the great disparity in ^1^H transverse relaxation times (T_2_) between water and protons within the protein, where ^1^H signal persists for a much longer time within the water bath. Thus, using a T_2_ filter we can actively select the ^1^H signal originating from the surrounding water. This signal is transferred to the protein *via* spin diffusion. This polarization transfer follows a characteristic buildup curve obeying a rate equation we adapted previously (Borcik et al., 2020; Luo and Hong, 2010; Najbauer et al., 2019). Representative buildup curves are depicted in Figure 1A. These buildup curves exhibit a good overall fit to our derived rate equation (Eq. 1) (Borcik et al., 2020). As depicted in Figure 1B in red, 4 ms of ^1^H_water_-^1^H_protein_ mixing is a good representation of solvent-exposed residues. To better understand the water accessible surface, we also acquired a spectrum with 16 ms of ^1^H-^1^H spin diffusion. The contoured difference in these spectra is depicted in Figure 1C. At 16 ms of ^1^H_water_-^1^H_protein_ spin-diffusion more embedded parts of protein appear in the spectra, consistent with fit buildup curves presented in Figure 1A. With 16 ms of ^1^H_water_-^1^H_protein_ mixing we observe most resonances in a standard DARR spectrum without a T_2_ filter, further indicating large spin-diffusion coverage (Supplementary Figure S1).

$$M_{p}\left(t_{m}\right)=M_{w}\left(\frac{2R_{p}}{R_{1p}+2R_{p}-R_{1w}}\right)\left(e^{-R_{1w}t_{m}}-e^{-\left(R_{1p}+2R_{p}\right)t_{m}}\right)$$ (1)

In Eq. 1 the p index specifies protein and w specifies water. M is magnetization on the specified chemical species at mixing time t_m_, and R is the rate of longitudinal cross relaxation for the specified species.

**FIGURE 2:**
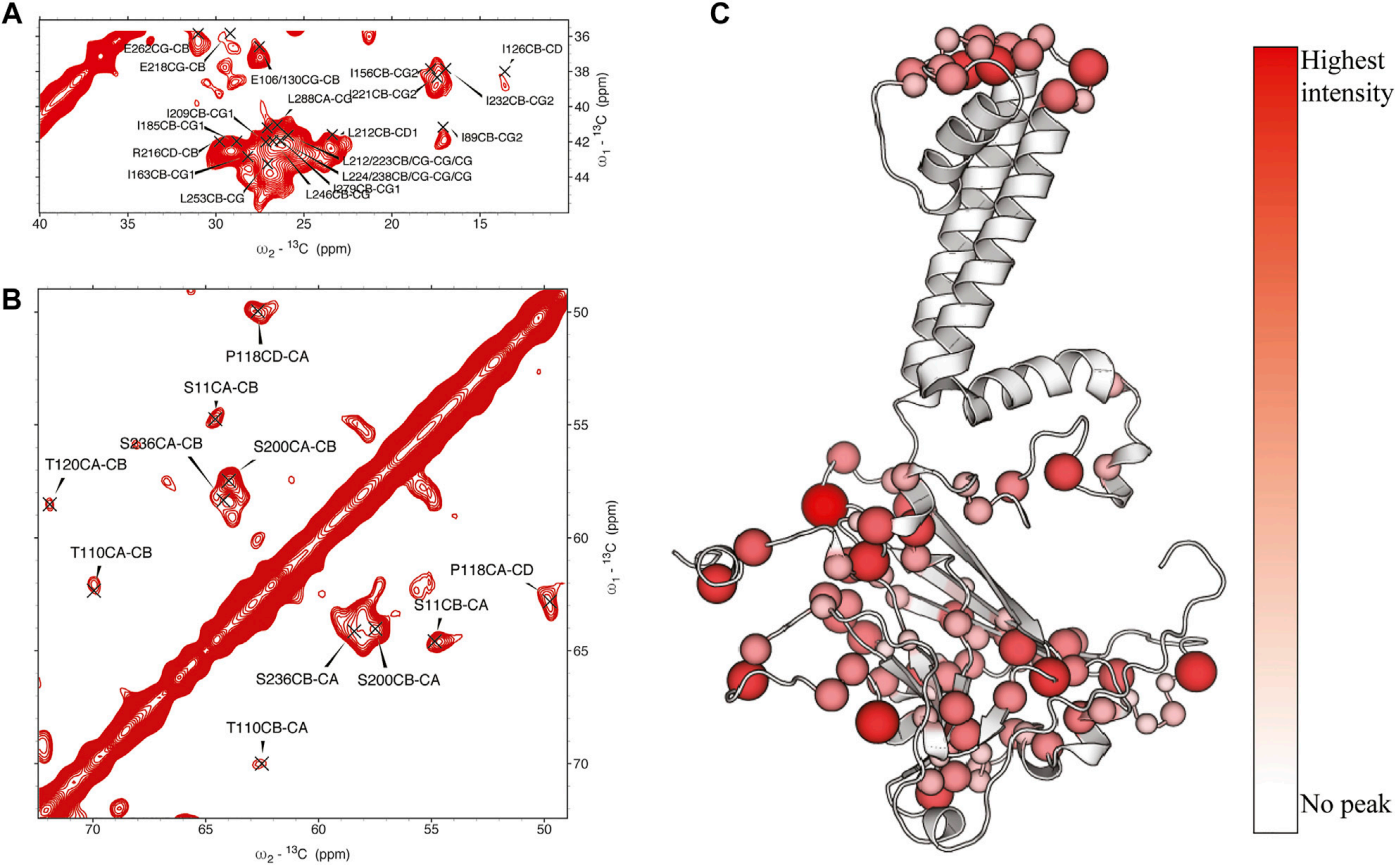
**(A, (B)** assigned regions of ^13^C-^13^C water-edited spectrum with 4 ms ^1^H_water_-^1^H_protein_ mixing, **(C)** position of assigned residues on the structure of KirBac1.1 with relative intensity represented in color and size of spheres.

^***13***^ ***C-*** ^***13***^ ***C-*** ^***13***^ ***C 3D spectrum.*** We obtained sparse distance restraints for tertiary and quaternary structure from a CCC 3D spectrum with 50 and 500 ms of DARR mixing during the first and second mixing periods. The 3D cross peaks were assigned based upon our reported 3D chemical shift assignments. Only completely unambiguous cross peaks were assigned, providing 54 intra subunit distances and 3 inter subunit distances. Given that the sample was uniformly ^13^C enriched, this limited number of distances was expected. More extensive distance assignments would require significantly less isotopic enrichment to provide the needed resolution.

In KirBac1.1, we consistently found the best ^1^H_water_-^1^H_protein_ mixing time for surface residues to be 4 ms. We were able to assign many sites in these spectra (Figure 2). Initially, 51 unambiguous water-edited peaks were assigned based upon our chemical shift assignments for this state of the protein. After multiple iterations of structure refinement, the initial structures helped us to assign an additional 187 ambiguous peaks for a total of 238 solvent-accessibility restraints (Supplementary Figure S2) as described below. However, many solvent accessible peaks, especially methyl groups, remained too degenerate for reasonable assignment. However, three- and four-dimensional versions of these pulse sequences may resolve this ambiguity in even more challenging membrane protein systems.

**FIGURE 3:**
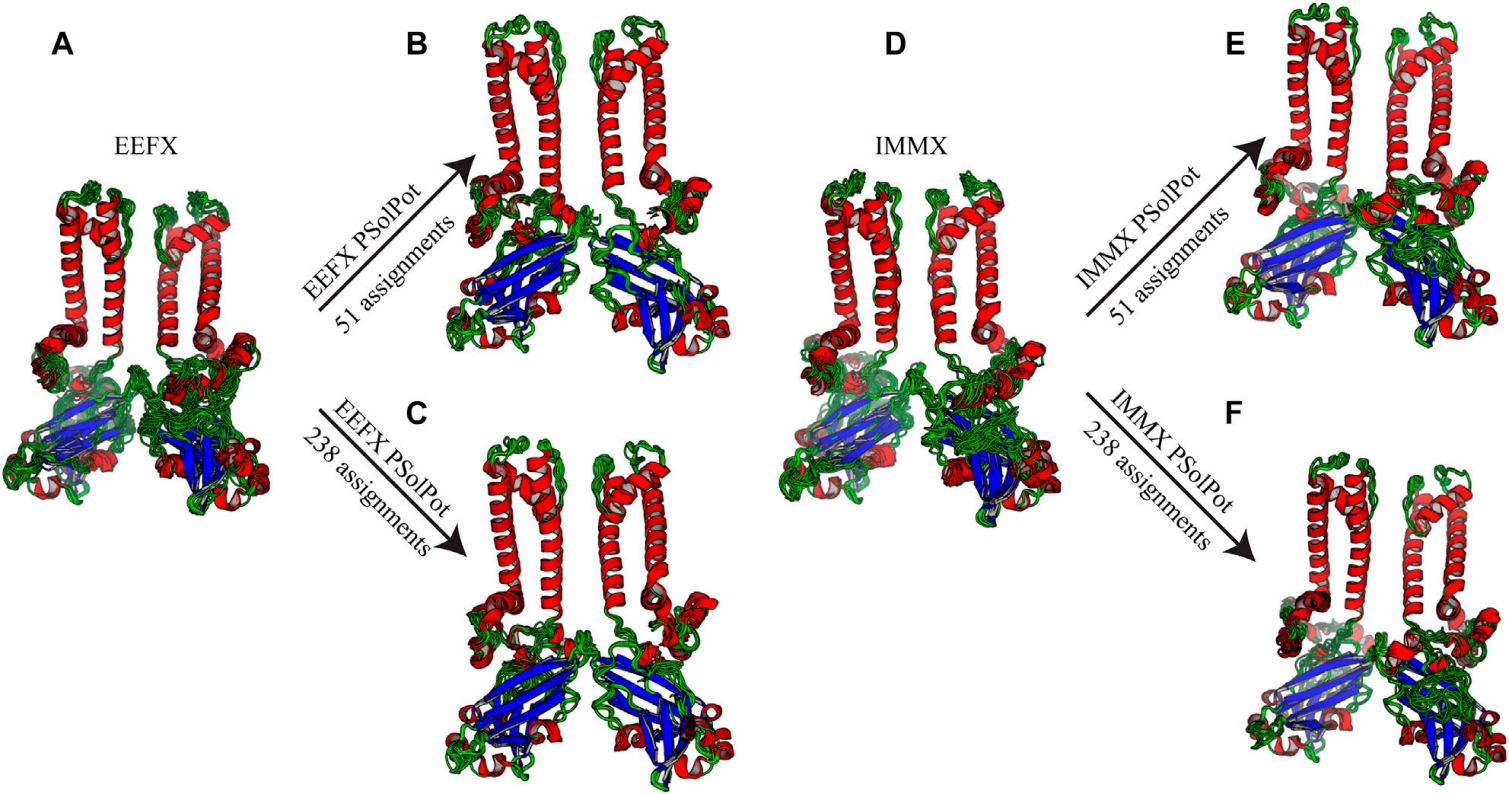
Ensemble of ten lowest structure in each step of calculation **(A)** No water-edited restraints with EEFx potential term, **(B)** 51 unambiguous water-edited restraints with EEFx potential, **(C)** 238 ambiguous and unambiguous water-edited restraints with EEFx potential term, **(D)** No water-edited restraints with IMMx potential term, **(E)** 51 unambiguous water-edited restraints with IMMx potential term, **(F)** 238 ambiguous and unambiguous water-edited restraints with IMMx potential term.

***Initial structural calculations.*** We started our structure refinement process by generating structures using dihedral angles and distance information in Xplor-NIH. The protocol described above in which the PSolPot term was not used in the initial phase was necessitated by difficulties the term has in representing the very extended structures obtained during initial randomization. Using our previously reported ^13^C chemical shift assignments, we determined backbone dihedral angles in TALOSN. The prediction resulted in 502 dihedral angle restraints (φ, ψ). In the first step of structural refinement, 100 structures were generated using Xplor-NIH version 3.2.9 with 502 SSNMR dihedral angles, 54 intra- and 3 inter-subunit SSNMR unambiguous distances, the hydrogen bond potential of mean force (HBPot), and the EEFx potential adapted from CHARMM22 (MacKerell et al., 1998; Tian et al., 2015). The ensemble of the 10 lowest energy structures is presented in Figure 3A. An additional 100 structures were generated with identical restraints, but with the addition of the IMMx function to the EEFx potential with membrane parameters of 27.0 for POPC membrane thickness, profileN set to 2, and the delectric screening value or A parameter to 0.85 (calculations that lack the IMMx function are simply called EEFx and the calculations with IMMx added to EEFx potential function are called IMMx). This ensemble is presented in Figure 3D. As shown in Tables 1, 2, the calculated pairwise RMSD (pwRMSD) *via* VMD-Xplor for the first step of this structure calculation without water-edited restraints, are 2.4 ± 1 Å and 2.2 ± 0.5 Å for backbone residues 1 to 301 of the EEFx and IMMx calculations respectively. The pwRMSD for the backbone of the well-ordered regions of the protein (residues 40–282) improves to 2.2 ± 1 and 2 ± 0.5 for the EEFx and IMMx calculations respectively. Although these pwRMSD are acceptable for this level of experimental dihedral angle and distance restraints, there is significant room for improvement. This improvement in the quality of the calculated structures shows the importance of water-edited restraint usage for the structure calculation.

**TABLE 1:** Structural statistics for calculations with EEFx potential.

Structure statistics	No PSolPot term	51 PSolPot restraints	238 PSolPot restraints	223 PSolPot restraints
Violations (mean ± σ)				
Bond lengths (A°)	0.0101 ± 0.0003	0.009 ± 0.0	0.009 ± 0.0	0.009 ± 0.0
Bond angles (°)	1.21 ± 0.04	1.196 ± 0.008	1.199 ± 0.007	1.195 ± 0.008
Improper (°)	1.21 ± 0.06	1.05 ± 0.01	1.06 ± 0.01	1.06 ± 0.01
Pairwise r.m.s.d. (A°)				
Heavy atoms (1–301)	3.1 ± 0.8	2 ± 0.2	2 ± 0.1	1.9 ± 0.1
Backbone (1–301)	2.4 ± 1	0.9 ± 0.2	0.9 ± 0.2	0.8 ± 0.2
Heavy atoms (40–282)	3.1 ± 0.7	1.8 ± 0.2	1.9 ± 0.2	1.8 ± 0.1
Backbone (40–282)	2.2 ± 1	0.7 ± 0.3	0.8 ± 0.2	0.7 ± 0.2
Ensemble backbone to crystal structure	3.1 ± 1.3	1.66 ± 0.05	1.68 ± 0.05	1.66 ± 0.05

**TABLE 2:** Structural statistics for IMMx potential.

Structure statistics	No PSolPot term	51 PSolPot restraints	238 PSolPot restraints	223 PSolPot restraints
Violations (mean ± σ)				
Bond lengths (A°)	0.0101 ± 0.0003	0.009 ± 0.0	0.009 ± 0.0	0.009 ± 0.0
Bond angles (°)	1.23 ± 0.03	1.24 ± 0.01	1.240 ± 0.006	1.241 ± 0.007
Improper (°)	1.16 ± 0.06	1.12 ± 0.04	1.12 ± 0.03	1.10 ± 0.02
Pairwise r.m.s.d. (A°)				
Heavy atoms (1–301)	3.1 ± 0.4	2 ± 0.1	2.09 ± 0.09	2.1 ± 0.1
Backbone (1–301)	2.2 ± 0.5	0.9 ± 0.2	1.0 ± 0.1	1.0 ± 0.2
Heavy atoms (40–282)	2.9 ± 0.4	1.80 ± 0.08	1.84 ± 0.09	1.83 ± 0.08
Backbone (40–282)	2.0 ± 0.5	0.7 ± 0.1	0.7 ± 0.1	0.7 ± 0.1
Ensemble backbone to crystal structure	2.1 ± 0.1	1.71 ± 0.05	1.75 ± 0.06	1.75 ± 0.07

**Water-accessibility restraints.** Previous applications of solvent accessibility as a structure refinement tool utilized solution based paramagnetic relaxation enhancement (sPRE). Generally, paramagnetic relaxation enhancement (PRE) results from the coupling between a magnetically active nucleus and an unpaired electron. The electron may be a stable radical or metal. This unpaired electronic spin may be bound to the protein or free in solution. This interaction has r^−6^ range dependence. Recently, soluble paramagnetic probes gained popularity. When these moieties contact the surface of the protein, they introduce the sPRE which can thus be tied to solvent accessible surface (S_Acc_). Surface accessibility restraints were initially incorporated in Xplor-NIH using an empirical expression involving distances to neighboring nuclei, and it was shown to qualitatively represent water-protein interactions in solution and solvent PRE data (Wang et al., 2012). Wang *et al.* found that S_Acc_ can be calculated with a linear equation, where the slope and intercept is a unique function of a specific protein’s topology. More recently (Gong et al., 2018; Kooshapur et al., 2018), a more quantitative representation of solvent PRE data has been developed, where the observable is represented by Eq. 2. For sPREs this expression is approximate, with the quantitative relationship between Eq. 2 and solvent PRE being somewhat more complicated (Okuno et al., 2020), and yet this formulation has been employed with some success. In this vein, our residue-based water-edited SSNMR-derived surface area data are fit to values computed from molecular structure using Eq. 2. In keeping with the qualitative nature of the representation, the corresponding Xplor-NIH energy term depends only on the correlation between the two quantities (Gong et al., 2018). Gong *et al.* and Kooshapur *et al* proposed a grid search algorithm to determine the accessible surface. This included a protein surface integral that can be written in form of a tessellation composed of triangular patches (Eq. 2). In Eq. 2, k' is a constant prefactor, n is the outward-facing distance normal surface, and r is the distance from this surface to a nucleus:

$$\Gamma_{sPRE}=\frac{-k^{\prime }}{9}\sum_{i}a_{i}n_{i}.\frac{r_{i}}{{\left|r_{i}\right|}^{6}}$$ (2)

They incorporated these concepts into sPRE module and energy potential (PSolPot) to include sPRE data in Xplor-NIH simulated annealing calculations. This potential was shown to be quite effective in direct structure refinement (Gong et al., 2018; Kooshapur et al., 2018).

We now present a new application of the PSolPot potential function to refine protein structures using water-edited SSNMR spectroscopy derived restraints. Water-edited SSNMR identifies the accessible surface of the protein with a similar r^−6^ distance dependence. As described above, previous studies found that the overall surface area of the water-protein interface can be expressed by Eq. 3

$$S_{Acc}=V_{P}\sqrt{\frac{\pi }{D_{eff}t_{m}^{s}}}$$ (3)

Where S_Acc_ is the surface area of the water-protein interface, $t_{m}^{s}$ is the time of mixing until saturation, V_P_ is the volume of the protein, and D_eff_ is the effective diffusion parameter. This equation provides a global picture rather than a site-specific view of the water-protein interface. Following Andreas et al. (Najbauer et al., 2019), we previously found the water-to-protein polarization transfer could be defined by a longitudinal cross relaxation-dependent rate equation stated above (Eq. 1).

It has been long known (Bloembergen et al., 1948) that the relaxation term for longitudinal cross relaxation depends on the ^1^H-^1^H dipolar coupling that has the form of equation 4 from dipolar alphabet.

$${\langle H_{loc}^{2}\rangle }_{Av}=\;\frac{1}{3}\;\gamma^{2}\hbar^{2}I\left(I+1\right)\sum_{j}{\left(1-3\cos^{2}\theta_{ij}\right)}^{2}r_{ij}^{-6}$$ (4)

Thus, because of the similar r^−6^ dependence, we found the PSolPot potential could accommodate our restraints after modification.

**FIGURE 4:**
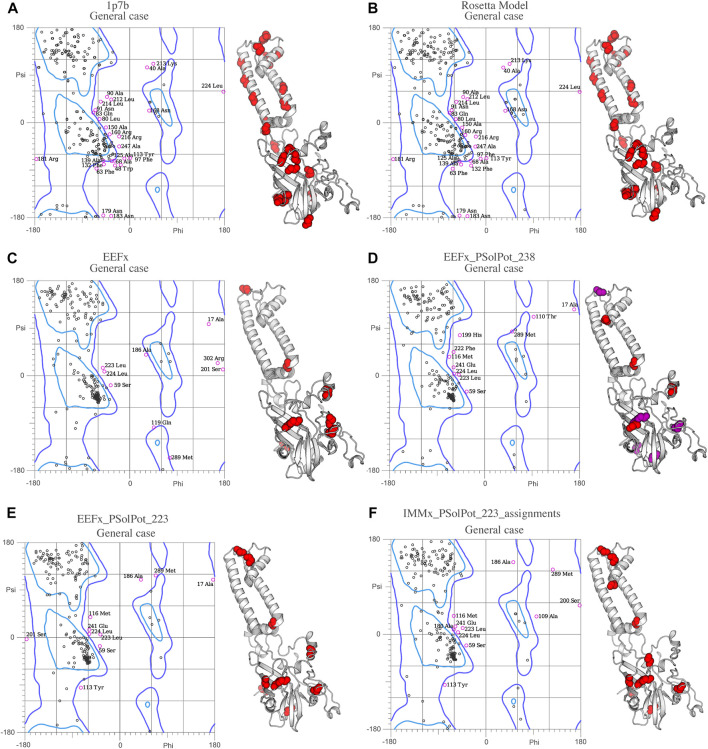
Ramachandran space of **(A)** Crystal structure 1p7b, **(B)** Initial Rosetta model, **(C)** Lowest structure of EEFx run, **(D)** The lowest energy structure of PSolPot, residues in magenta are the outlier residues in Ramachandran space that has been deleted in the last round of structure calculation via PSolPot. **(E)** The lowest energy structure without Ramachandran outlier in PSolPot list with EEFx potential term. **(F)** The lowest energy structure without Ramachandran outlier in PSolPot list with IMMx potential term.

As shown in Figure 2, our extensive chemical shift assignments of water-edited spectra provide restraints for nearly half the protein (full assignments of the aliphatic region are shown in Supplementary Figure S2). Because PSolPot is a correlation function that fits the water accessible surface, the relative signal intensity of each peak can be used as the data input for structural refinement. The chemical shift assignments of the water-edit spectra were performed in two rounds. In the first round, the integrated intensity of resolved unambiguous peaks were used for structure refinement. These 51 assignments were used to refine two sets of 100 structures starting from the 10 lowest energy structures of the EEFx (Figure 3B) and IMMx (Figure 3E) calculations respectively. In the second round, we included the integrated intensity of all possible assignments, corresponding to 238 total PSolPot restraints. This provided two additional sets of 100 structures starting from the same PSolPot-free EEFx (Figure 3C) and IMXx (Figure 3F) ensembles. We then determined the bbRMSD and judged the quality in MolProbity. This indicated the overall structural quality slightly diminished relative to the PSolPot ensemble with only unambiguous restraints (Table 3 and Figure 4). We then deleted 6 Ramachandran outliers and their neighboring residues and repeated the calculations with only 223 PSolPot restraints. This slightly improved the overall structural quality.

**TABLE 3:** Comparison of structure quality performed on MolProbity for the generated structures to initial structures.

	Favored rotamers (%)	Ramachandran favored (%)	Rama distribution Z-score (less than 2 is the goal)
Crystal structure 1p7b	66.2	60.9	−6.7 ± 0.3
Rosetta Model	74.3	66.5	−6.0 ± 0.2
Lowest generated EFFx structure	82.9	85.2	−1.7 ± 0.2
Lowest EEFx-PSolPot 51 assignments	88.1	87.6	−0.8 ± 0.2
Lowest EEFx-PSolPot 238 Assignments	87.0	86.4	−1.4 ± 0.2
Lowest EEFx-PSolPot 223 Assignments	91.5	87.3	−0.8 ± 0.2
Lowest IMMx structure	85.5	84.6	−1.6 ± 0.2
Lowest IMMx-PSolPot 51 assignments	89.6	87.6	−0.7 ± 0.2
Lowest IMMx-PSolPot 238 Assignments	91.1	85.2	−0.5 ± 0.2
Lowest IMMx-PSolPot 223 Assignments	92.6	85.5	−0.7 ± 0.2

We judged the internal consistency of all four final structural ensembles using heavy-atom RMSD, and backbone RMSD. We also judged their objective quality *via* rotameric, Ramachandran, and Z-score analysis in MolProbity. The statistical summary of all four stages of simulated annealing with EEFx are provided in Table 1. These calculations include the ensemble with only EEFx, the addition of 51 unambiguous assignments, 238 assignments (ambiguous and unambiguous), and finally 223 assignments (ambiguous and unambiguous without Ramachandran outliers). In Table 2 the same statistics are listed after IMMx is included in the calculation. As mentioned above, the addition of the unambiguous water-edited restraints in structure calculation improved the structural quality dramatically. Using the EEFx forcefield and the 51 unambiguous restraints, the pwRMSD for the protein backbone improved from 2.4 ± 1 Å to 0.9 ± 0.2 Å (Table 1) for residues 1 to 301. In the well-ordered regions, residues 40 to 282, the pwRMSD improved from 2.2 ± 1 Å to 0.7 ± 0.3 Å. When the IMMx membrane potential is added to the calculation the pwRMSD improved from 2.2 ± 0.5 Å to 0.9 ± 0.2 Å for the first 301 residues, and from 2.0 ± 0.5 Å to 0.7 ± 0.1 Å for the well-ordered regions (Table2). In most cases the utilization of IMMx produces improvements relative to EEFx within the hydrophobic region of the protein. The addition of unambiguous water-edited restraints did not result in a significant improvement in the structure, where the pwRMSD slightly diminished. Deleting identified Ramachandran violators only improved the pwRMSD slightly.

Overall, the objective structural quality improved with the addition of EEFx, IMMx, and PSolPot restraints as judged by MolProbity. As stated above, the 1p7b crystal structure lacks 75 residues (22.5% of the residues in WT-KirBac1.1 sequence). Our initial Rosetta model includes all 75 residues missing from 1p7b. Out of the 75 residues missing in the X-ray crystal structure, we have experimental SSNMR restraints for 51 residues. The final 24 residues of the protein remain unrestrained. Despite the incomplete information on end of the C-terminus of protein, the best structural ensembles possess up to 92.6% favored rotameric scores and up to 87.6% of residues occupy most favored regions of Ramachandran space. Ramachandran plots and the location of Ramachandran outliers is depicted in Figure 4. As shown in Table 3, the inclusion of EEFx and the 51 unambiguous PSolPot restraints dramatically improved the structural quality compared to both the 1p7b X-ray structure and the starting Rosetta model. The population of residues in favored Ramachandran and rotameric space increases from 66.5 to 74.3% to 85.2 and 82.9% when EEFx terms are included. The addition of 51 PSolPot restraints further improves these statistics to 87.6 and 88.1% favored occupancy. As further depicted in Table 3 and Figure 4, the inclusion of all possible PSolPot restraints does improve upon structural ensembles without these restraints, but slightly deteriorates, indicating that PSolPot is very capable of improving structures with a set of high-quality but relatively sparse restraints, compared to a much longer list of lower-quality information. However, after fully analyzing the structures solved with 238 restraints, and comparing Ramachandran violators to overlapped regions of solvent accessible spectra, we deleted several Ramachandran space violators. This improved the favored rotamer percentage and favored Ramachandran percentage to 91.5 and 87.6%. This further indicates that PSolPot is best implemented with high-quality rather than high-quantity restraints. After significant data quality control, only marginal improvement over the unambiguous structure is observed.

## Conclusion

Structural refinement of KirBac1.1 was performed using predicted dihedral angles from SSNMR chemical shift assignments, unambiguous distance restraints, and SSNMR water-edited spectroscopy. Calculations were carried out using Xplor-NIH version 3.2.9. The statistical comparison of the 10 lowest energy structures solved with water-edited SSNMR restraints supplied to the PSolPot potential improved both the backbone and all heavy-atom RMSDs relative to ensembles without these restraints. The pair-wise bbRMSD improved from 2.4 Å to 0.9 Å after including PSolPot in the calculation, which is a 62.5% improvement. However, it is clear, that given the nature of the grid search matrix algorithm inherent to PSolPot, relatively sparse but high-quality restraints can create a significant improvement in protein quality. However, including of less-resolved sites in the protein will require significant further analysis. Yet, it is clear even incomplete water-accessibility, and perhaps lipid accessibility, can be a powerful means to structural improvement. Given the difficulty and complexity in solving the structures of transmembrane proteins by NMR, this technique provides a new powerful means to solve and refine the structures of these proteins, which are fundamental to human health. Given the wide application of water-edited SSNMR, this technique could soon reach wide acceptance. In addition, it is clear this method is compatible with the implicit lipid and water models within Xplor-NIH. It was previously shown that EEFx (Tian et al., 2014; Tian et al., 2015) and IMMx (Tian et al., 2017) were powerful means for *de novo* solution of monomeric membrane proteins. Based upon our results, these forcefields are also applicable to membrane protein oligomers provided the appropriate parameter adjustments during annealing and refinement respectively. In future studies the application of this technique for *de novo* structure determination can be tested.

## Data Availability

The datasets presented in this study can be found in online repositories. The names of the repository/repositories and accession number(s) can be found below: https://doi.org/10.13018/BMR50135, 50135.

## References

[B1] Ader C. Schneider R. Seidel K. Etzkorn M. Becker S. Baldus M. (2009). Structural Rearrangements of Membrane Proteins Probed by Water-Edited Solid-State NMR Spectroscopy. J. Am. Chem. Soc. 131, 170–176. 10.1021/ja806306e 19063626

[B2] Amani R. Borcik C. G. Khan N. H. Versteeg D. B. Yekefallah M. Do H. Q. (2020). Conformational Changes upon Gating of KirBac1.1 into an Open-Activated State Revealed by Solid-State NMR and Functional Assays. Proc. Natl. Acad. Sci. USA 117, 2938–2947. 10.1073/pnas.1915010117 31980523 PMC7022178

[B3] Andrew E. R. Bradbury A. Eades R. G. (1958). Nuclear Magnetic Resonance Spectra from a Crystal Rotated at High Speed. Nature 182, 1659. 10.1038/1821659a0

[B4] Bermejo G. A. Schwieters C. D. (2018). Protein Structure Elucidation from NMR Data with the Program Xplor-NIH. Methods Mol. Biol. 1688, 311–340. 10.1007/978-1-4939-7386-6_14 29151215 PMC6771931

[B5] Bhate M. P. Wylie B. J. Thompson A. Tian L. Nimigean C. McDermott A. E. (2013). Preparation of Uniformly Isotope Labeled KcsA for Solid State NMR: Expression, Purification, Reconstitution into Liposomes and Functional Assay. Protein Expr. Purif. 91, 119–124. 10.1016/j.pep.2013.07.013 23916531 PMC3805054

[B6] Bloembergen N. Purcell E. M. Pound R. V. (1948). RELAXATION EFFECTS IN NUCLEAR MAGNETIC RESONANCE ABSORPTION. Phys. Rev. 73, 679–712. 10.1103/PhysRev.73.679

[B7] Borcik C. G. Versteeg D. B. Amani R. Yekefallah M. Khan N. H. Wylie B. J. (2020). The Lipid Activation Mechanism of a Transmembrane Potassium Channel. J. Am. Chem. Soc. 142, 14102–14116. 10.1021/jacs.0c01991 32702990 PMC8281327

[B8] Cheng W. W. L. Enkvetchakul D. Nichols C. G. (2009). KirBac1.1: It's an Inward Rectifying Potassium Channel. J. Gen. Physiol. 133, 295–305. 10.1085/jgp.200810125 19204189 PMC2654083

[B9] Clarke O. B. Caputo A. T. Hill A. P. Vandenberg J. I. Smith B. J. Gulbis J. M. (2010a). Domain Reorientation and Rotation of an Intracellular Assembly Regulate Conduction in Kir Potassium Channels. Cell 141, 1018–1029. 10.1016/j.cell.2010.05.003 20564790

[B10] Clarke O. B. Caputo A. T. Hill A. P. Vandenberg J. I. Smith B. J. Gulbis J. M. (2010b). Domain Reorientation and Rotation of an Intracellular Assembly Regulate Conduction in Kir Potassium Channels. Cell 141, 1018–1029. 10.1016/j.cell.2010.05.003 20564790

[B11] Enkvetchakul D. Bhattacharyya J. Jeliazkova I. Groesbeck D. K. Cukras C. A. Nichols C. G. (2004). Functional Characterization of a Prokaryotic Kir Channel. J. Biol. Chem. 279, 47076–47080. 10.1074/jbc.C400417200 15448150 PMC8629170

[B12] Enkvetchakul D. Jeliazkova I. Bhattacharyya J. Nichols C. G. (2007). Control of Inward Rectifier K Channel Activity by Lipid Tethering of Cytoplasmic Domains. J. Gen. Physiol. 130, 329–334. 10.1085/jgp.200709764 17698595 PMC2151642

[B13] Fung B. M. Khitrin A. K. Ermolaev K. (2000). An Improved Broadband Decoupling Sequence for Liquid Crystals and Solids. J. Magn. Reson. 142, 97–101. 10.1006/jmre.1999.1896 10617439

[B14] Gong Z. Schwieters C. D. Tang C. (2018). Theory and Practice of Using Solvent Paramagnetic Relaxation Enhancement to Characterize Protein Conformational Dynamics. Methods 148, 48–56. 10.1016/j.ymeth.2018.04.006 29656079 PMC6133729

[B16] Hornig S. Ohmert I. Trauner D. Ader C. Baldus M. Pongs O. (2013). Tetraphenylporphyrin Derivative Specifically Blocks Members of the Voltage-Gated Potassium Channel Subfamily Kv1. Channels 7, 473–482. 10.4161/chan.25848 24722265 PMC4042482

[B17] Huang P.-S. Ban Y.-E. A. Richter F. Andre I. Vernon R. Schief W. R. (2011). RosettaRemodel: a Generalized Framework for Flexible Backbone Protein Design. PLoS One 6, e24109. 10.1371/journal.pone.0024109 21909381 PMC3166072

[B18] Kooshapur H. Schwieters C. D. Tjandra N. (2018). Conformational Ensemble of Disordered Proteins Probed by Solvent Paramagnetic Relaxation Enhancement (sPRE). Angew. Chem. Int. Ed. 57, 13519–13522. 10.1002/anie.201807365 PMC6396310 30125451

[B19] Kumashiro K. K. Schmidt-Rohr K. Murphy O. J. Ouellette K. L. Cramer W. A. Thompson L. K. (1998). A Novel Tool for Probing Membrane Protein Structure: Solid-State NMR with Proton Spin Diffusion and X-Nucleus Detection. J. Am. Chem. Soc. 120, 5043–5051. 10.1021/ja972655e

[B20] Kuo A. Gulbis J. M. Antcliff J. F. Rahman T. Lowe E. D. Zimmer J. (2003). Crystal Structure of the Potassium Channel KirBac1.1 in the Closed State. Science 300, 1922–1926. 10.1126/science.1085028 12738871

[B21] Li S. Su Y. Luo W. Hong M. (2010). Water−Protein Interactions of an Arginine-Rich Membrane Peptide in Lipid Bilayers Investigated by Solid-State Nuclear Magnetic Resonance Spectroscopy. J. Phys. Chem. B 114, 4063–4069. 10.1021/jp912283r 20199036 PMC2853767

[B22] Linder T. Wang S. Zangerl-Plessl E.-M. Nichols C. G. Stary-Weinzinger A. (2015). Molecular Dynamics Simulations of KirBac1.1 Mutants Reveal Global Gating Changes of Kir Channels. J. Chem. Inf. Model. 55, 814–822. 10.1021/acs.jcim.5b00010 25794351 PMC4415035

[B23] Lowe I. J. (1959). Free Induction Decays of Rotating Solids. Phys. Rev. Lett. 2, 285–287. 10.1103/physrevlett.2.285

[B24] Luo W. Hong M. (2010). Conformational Changes of an Ion Channel Detected through Water−Protein Interactions Using Solid-State NMR Spectroscopy. J. Am. Chem. Soc. 132, 2378–2384. 10.1021/ja9096219 20112896 PMC2829254

[B25] Maciejewski M. W. Schuyler A. D. Gryk M. R. Moraru I. I. Romero P. R. Ulrich E. L. (2017). NMRbox: A Resource for Biomolecular NMR Computation. Biophysical J. 112, 1529–1534. 10.1016/j.bpj.2017.03.011 PMC5406371 28445744

[B26] MacKerell A. D. Bashford D. Bellott M. Dunbrack R. L. Evanseck J. D. Field M. J. (1998). All-atom Empirical Potential for Molecular Modeling and Dynamics Studies of Proteins. J. Phys. Chem. B 102, 3586–3616. 10.1021/jp973084f 24889800

[B27] Mandala V. S. Williams J. K. Hong M. (2018). Structure and Dynamics of Membrane Proteins from Solid-State NMR. Annu. Rev. Biophys. 47, 201–222. 10.1146/annurev-biophys-070816-033712 29498890 PMC6312106

[B28] Najbauer E. E. Movellan K. T. Schubeis T. Schwarzer T. Castiglione K. Giller K. (2019). Probing Membrane Protein Insertion into Lipid Bilayers by Solid‐State NMR. ChemPhysChem 20, 302–310. 10.1002/cphc.201800793 30452110

[B29] Nilges M. Clore G. M. Gronenborn A. M. (1988). Determination of Three-Dimensional Structures of Proteins from Interproton Distance Data by Hybrid Distance Geometry-Dynamical Simulated Annealing Calculations. FEBS Lett. 229, 317–324. 10.1016/0014-5793(88)81148-7 3345845

[B30] Okuno Y. Szabo A. Clore G. M. (2020). Quantitative Interpretation of Solvent Paramagnetic Relaxation for Probing Protein-Cosolute Interactions. J. Am. Chem. Soc. 142, 8281–8290. 10.1021/jacs.0c00747 32286812 PMC7372007

[B31] Paynter J. J. Andres-Enguix I. Fowler P. W. Tottey S. Cheng W. Enkvetchakul D. (2010). Functional Complementation and Genetic Deletion Studies of KirBac Channels. J. Biol. Chem. 285, 40754–40761. 10.1074/jbc.M110.175687 20876570 PMC3003375

[B32] Pines A. Gibby M. G. Waugh J. S. (1972). Proton‐Enhanced Nuclear Induction Spectroscopy. A Method for High Resolution NMR of Dilute Spins in Solids. J. Chem. Phys. 56, 1776–1777. 10.1063/1.1677439

[B33] Radoicic J. Lu G. J. Opella S. J. (2014). NMR Structures of Membrane Proteins in Phospholipid Bilayers. Quart. Rev. Biophys. 47, 249–283. 10.1017/S0033583514000080 PMC4153756 25032938

[B34] Renault M. Tommassen-van Boxtel R. Bos M. P. Post J. A. Tommassen J. Baldus M. (2012). Cellular Solid-State Nuclear Magnetic Resonance Spectroscopy. Proc. Natl. Acad. Sci. 109, 4863–4868. 10.1073/pnas.1116478109 22331896 PMC3323964

[B35] Schubeis T. Le Marchand T. Andreas L. B. Pintacuda G. (2018). H Magic-Angle Spinning NMR Evolves as a Powerful New Tool for Membrane Proteins. J. Magn. Reson. 287, 140–152. 10.1016/j.jmr.2017.11.014 29413327

[B36] Schwieters C. D. Bermejo G. A. Clore G. M. (2020). A Three-Dimensional Potential of Mean Force to Improve Backbone and Sidechain Hydrogen Bond Geometry in Xplor-NIH Protein Structure Determination. Protein Sci. 29, 100–110. 10.1002/pro.3745 31613020 PMC6933865

[B37] Schwieters C. D. Bermejo G. A. Clore G. M. (2018). Xplor-NIH for Molecular Structure Determination from NMR and Other Data Sources. Protein Sci. 27, 26–40. 10.1002/pro.3248 28766807 PMC5734396

[B38] Schwieters C. D. Clore G. M. (2001). The VMD-XPLOR Visualization Package for NMR Structure Refinement. J. Magn. Reson. 149, 239–244. 10.1006/jmre.2001.2300 11318623

[B39] Shen Y. Bax A. (2013). Protein Backbone and Sidechain Torsion Angles Predicted from NMR Chemical Shifts Using Artificial Neural Networks. J. Biomol. NMR 56, 227–241. 10.1007/s10858-013-9741-y 23728592 PMC3701756

[B40] Su Y. Waring A. J. Ruchala P. Hong M. (2011). Structures of β-Hairpin Antimicrobial Protegrin Peptides in Lipopolysaccharide Membranes: Mechanism of Gram Selectivity Obtained from Solid-State Nuclear Magnetic Resonance. Biochemistry 50, 2072–2083. 10.1021/bi101975v 21302955 PMC3062705

[B41] Takegoshi K. Nakamura S. Terao T. (2001). - Dipolar-Assisted Rotational Resonance in Magic-Angle Spinning NMR. Chem. Phys. Lett. 344, 631–637. 10.1016/S0009-2614(01)00791-6 17166029

[B42] Tian Y. Schwieters C. D. Opella S. J. Marassi F. M. (2015). A Practical Implicit Membrane Potential for NMR Structure Calculations of Membrane Proteins. Biophysical J. 109, 574–585. 10.1016/j.bpj.2015.06.047 PMC4572468 26244739

[B43] Tian Y. Schwieters C. D. Opella S. J. Marassi F. M. (2014). A Practical Implicit Solvent Potential for NMR Structure Calculation. J. Magn. Reson. 243, 54–64. 10.1016/j.jmr.2014.03.011 24747742 PMC4037354

[B44] Tian Y. Schwieters C. D. Opella S. J. Marassi F. M. (2017). High Quality NMR Structures: a New Force Field with Implicit Water and Membrane Solvation for Xplor-NIH. J. Biomol. NMR 67, 35–49. 10.1007/s10858-016-0082-5 28035651 PMC5487259

[B45] Tran N. T. Mentink-Vigier F. Long J. R. (2020). Dynamic Nuclear Polarization of Biomembrane Assemblies. Biomolecules 10, 1246. 10.3390/biom10091246 PMC7565305 32867275

[B46] van Aalst E. Yekefallah M. Mehta A. K. Eason I. Wylie B. (2020). Codon Harmonization of a Kir3.1-KirBac1.3 Chimera for Structural Study Optimization. Biomolecules 10, 430. 10.3390/biom10030430 PMC7175280 32164257

[B47] Wang S. Alimi Y. Tong A. Nichols C. G. Enkvetchakul D. (2009). Differential Roles of Blocking Ions in KirBac1.1 Tetramer Stability. J. Biol. Chem. 284, 2854–2860. 10.1074/jbc.M807474200 19033439 PMC2631979

[B48] Wang S. Lee S.-J. Heyman S. Enkvetchakul D. Nichols C. G. (2012a). Structural Rearrangements Underlying Ligand-Gating in Kir Channels. Nat. Commun. 3, 617. 10.1038/ncomms1625 22233627 PMC4277880

[B49] Wang Y. Schwieters C. D. Tjandra N. (2012b). Parameterization of Solvent-Protein Interaction and its Use on NMR Protein Structure Determination. J. Magn. Reson. 221, 76–84. 10.1016/j.jmr.2012.05.020 22750253 PMC3405189

[B50] Williams C. J. Headd J. J. Moriarty N. W. Prisant M. G. Videau L. L. Deis L. N. (2018). MolProbity: More and Better Reference Data for Improved All-Atom Structure Validation. Protein Sci. 27, 293–315. 10.1002/pro.3330 29067766 PMC5734394

[B51] Wylie B. J. Do H. Q. Borcik C. G. Hardy E. P. (2016). Advances in Solid-State NMR of Membrane Proteins. Mol. Phys. 114, 3598–3609. 10.1080/00268976.2016.1252470

[B52] Zhou D. H. Kloepper K. D. Winter K. A. Rienstra C. M. (2006). Band-selective 13C Homonuclear 3D Spectroscopy for Solid Proteins at High Field with Rotor-Synchronized Soft Pulses. J. Biomol. NMR 34, 245–257. 10.1007/s10858-006-0026-6 16645815

