# Supplementary material for: Water Accessibility Refinement of the Extended Structure of KirBac1.1 in the Closed State
Source: Front Mol Biosci. 2021 Nov 30;8:772855. doi: 10.3389/fmolb.2021.772855 (PMC8669819; doi:10.3389/fmolb.2021.772855)
Supplement: Supplementary file 1 [file DataSheet1.pdf]

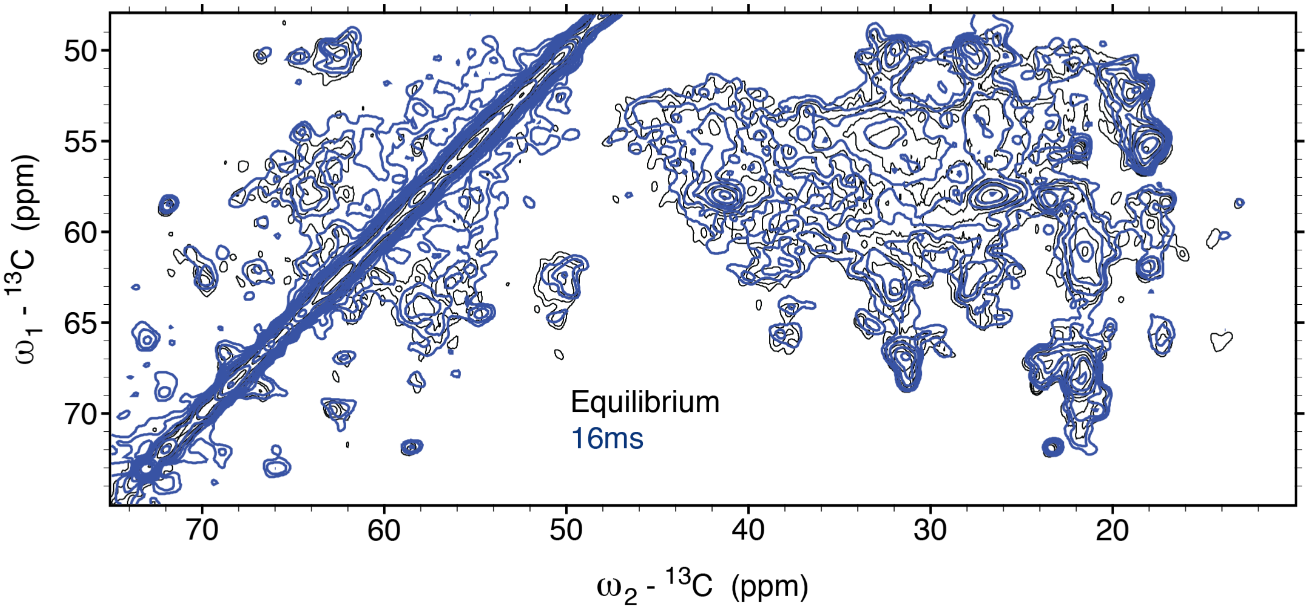

Figure S1. Overlay of water-edited 2D DARR experiment with 16 ms $^{1}H_{water}$-$^{1}H_{protein}$ mixing onto 2D DARR acquired without an H_2O T_2 filter.

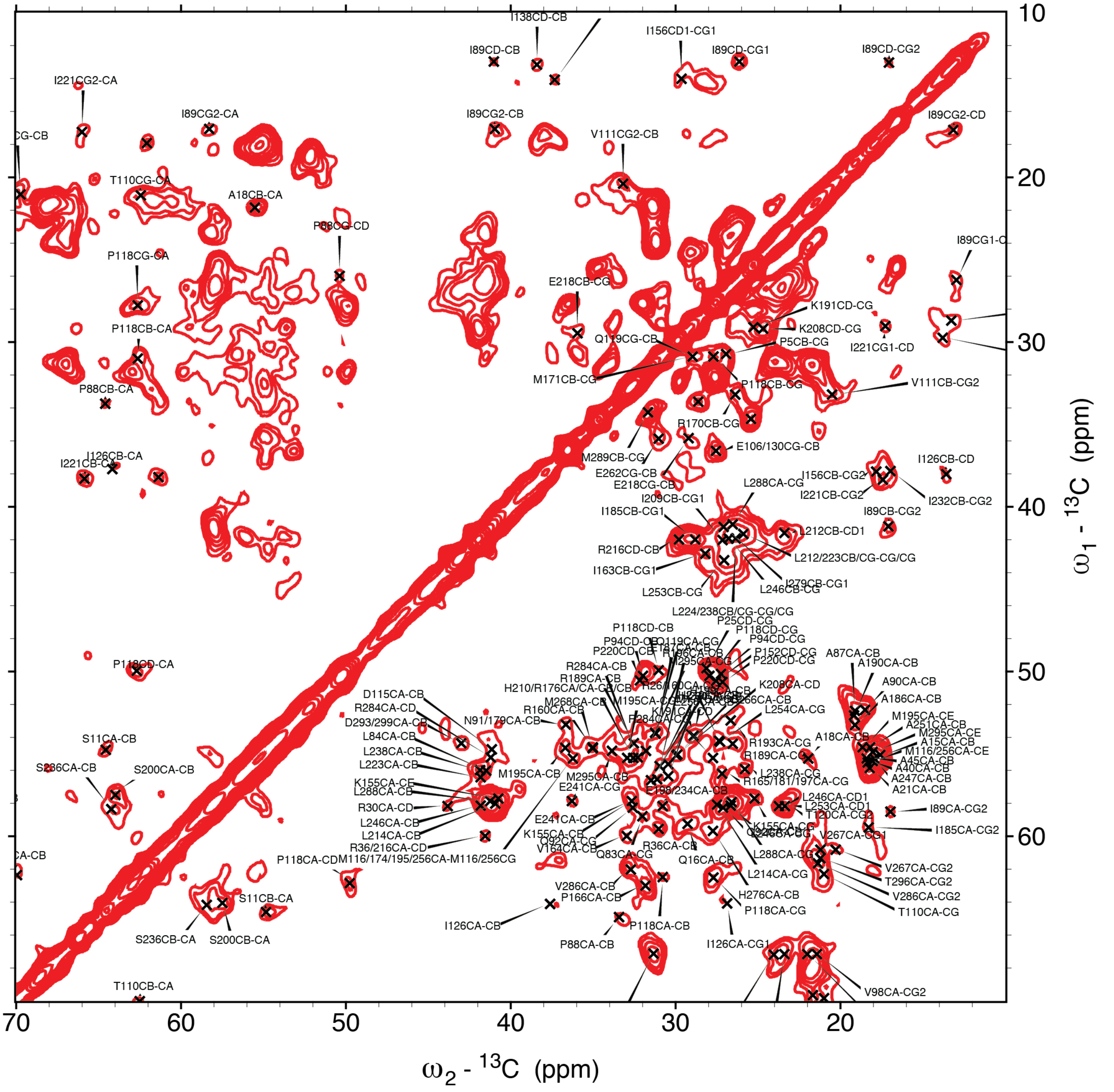

Figure S2. Fully assigned aliphatic region of water-edited 2D DARR spectrum with 4 ms $^{1}H_{water}$-$^{1}H_{protein}$ mixing.

Python script for Xplor-NIH for symmetry element

```
def init():
    """configures symSim as a SymSimulation
    """

    from atomSel import AtomSel
    from symSimulation import SymSimulation
    symSim = SymSimulation('symSim',
                           AtomSel("not pseudo"),
                           cloneFirst=False)

    from math import pi
    from vec3 import Vec3
    from mat3 import rotVector, Mat3
    for i,segid in enumerate( "A B C D".split() ):
        symSim.addCopy(rotVector(Vec3(0,0,1),i * pi/2),
                       segidSuffix=segid)
        pass

    return symSim
```

Python script for Xplor-NIH for first step of refinement with EEFx

```
import protocol

#import prePot

#xplor.requireVersion("2.4")


#

# this version refines from a reasonable model structure.

#

# CDS 2005/05/10

#



(opts,args) = xplor.parseArguments(["quick"]) # check for command-line typos


quick=False

for opt in opts:

    if opt[0]=="quick":  #specify -quick to just test that the script runs

        quick=True

        pass

    pass



numberOfStructures=100


if quick:
```

```
    numberOfStructures=1
    pass

# protocol module has many high-level helper functions.
#
import protocol
protocol.initRandomSeed(3421)   #explicitly set random seed

# INITIALIZE PARAMETERS AND TOPOLOGY.
import eefxPotTools
eefxPotTools.initEEFx()

#
# annealing settings
#

command = xplor.command

protocol.initParams("protein")

#
#protocol.genExtendedStructure("gb1_extended_%d.pdb" %
#                              protocol.initialRandomSeed())

import protocol
```

```
startPDB='Rosetta_tetramer_4k_I131C.pdb'
protocol.loadPDB(startPDB,deleteUnknownAtoms=True)


xplor.simulation.deleteAtoms("segid B C D")


from atomSelAction import SetProperty
AtomSel("all").apply( SetProperty("segmentName","") )


import symSimSetup
symSim = symSimSetup.init()
##generate distance restraints from the starting structure
if xplor.p_processID==0:
   from noePotTools import genRestraints
   open('distance_K.tbl','w').write(genRestraints("name O K and resid 108:116 401:404 ",
                                    distCutoff=6.0,
                                    upperBound=2.0,
                                    lowerBound=1.0)
                          )
   pass


#
# a PotList contains a list of potential terms. This is used to specify which
# terms are active during refinement.
#
from potList import PotList
```

```
potList = PotList()
crossTerms = PotList('cross')


# parameters to ramp up during the simulated annealing protocol
#
from simulationTools import MultRamp, StaticRamp, InitialParams


rampedParams=[]
highTempParams=[]


# compare atomic Cartesian rmsd with a reference structure
#  backbone and heavy atom RMSDs will be printed in the output
#  structure files
#
from posDiffPotTools import create_PosDiffPot
protomerNCS = create_PosDiffPot("ncs",
                           "name CA C O N and resid 46:150 161:281",
                           selection2="segid A and name CA C O N and resid 46:150 161:281",
                           pdbFile=startPDB)


protomerNCS.setScale( 5 )
protomerNCS.setUpperBound( 0 )
potList.append( protomerNCS )
##allowed to be up to 3 Angstroms from starting structure at the end of the
## calculation
#rampedParams.append( MultRamp(1,3, "protomerNCS.setUpperBound( VALUE )") )
```

```
# lists to hold force constant settings for various stages of
# structure calculation
from simulationTools import MultRamp, StaticRamp
hiTempParams = []
# Settings for annealing stage.
rampedParams = []

# set up NOE potential
noe=PotList('noe')
potList.append(noe)
from noePotTools import create_NOEPot
for (name,scale,file) in [('all',1,"KB11_POPC_Intra_distances.tbl"),
                          #add entries for additional tables
                          ]:
    pot = create_NOEPot(name,file)
    # pot.setPotType("soft") # if you think there may be bad NOEs
    pot.setScale(scale)
    noe.append(pot)
rampedParams.append( MultRamp(2,30, "noe.setScale( VALUE )") )


from noePotTools import create_NOEPot
Kdist = create_NOEPot("K-dist","distance_K.tbl")
potList.append(Kdist)
```

```
rampedParams.append( MultRamp(2,30, "Kdist.setScale( VALUE )") )



import simulation
simulation.makeCurrent( symSim )

## gyration volume term
##
#from gyrPotTools import create_GyrPot
#gyr = create_GyrPot("Vgyr",
#                    "resid 36:300") # selection should exclude disordered tails
#potList.append(gyr)
#rampedParams.append( MultRamp(.002,1,"gyr.setScale(VALUE)") )
# set up NOE potential
restraints=""
for resid in range(401,404):
   kAtoms= AtomSel(f"resid {resid} and name K*")
   from itertools import combinations
   for atom1,atom2 in combinations(kAtoms,2):
      restraints += f"assi (atom {atom1.segmentName()} {resid} K)\n"
      restraints += f"assi (atom {atom2.segmentName()} {resid} K) "
      restraints += " 2 2 0\n"
      pass
   pass
print( restraints)
Kaxis = create_NOEPot("K-axis",restraints=restraints)
```

```
potList.append(Kaxis)
rampedParams.append( MultRamp(2,30, "Kdist.setScale( VALUE )") )


from noePotTools import create_NOEPot
InterDist = create_NOEPot("InterDist","KB11_POPC_Inter_distances.tbl")
potList.append(InterDist)
rampedParams.append( MultRamp(2,30, "InterDist.setScale( 4*VALUE )") )


#noe=PotList('noe')
#potList.append(noe)
#from noePotTools import create_NOEPot
#for (name,scale,file) in [('all',1,"distance_K.tbl"),
#                          #('KB11_POPC_Intra_distances.tbl')
#                         #add entries for additional tables
#                         ]:
#    pot = create_NOEPot(name,file)
#    # pot.setPotType("soft") # if you think there may be bad NOEs#
#    pot.setScale(scale)
#    noe.append(pot)
#rampedParams.append( MultRamp(2,30, "noe.setScale( VALUE )") )


# HBPot - knowledge-based hydrogen bond term
#
from hbPotTools import create_HBPot
hb = create_HBPot('hb')
```

```
hb.setScale(2.5)
potList.append( hb )

#nonbonded term - EEFx
from eefxPotTools import create_EEFxPot, param_LK
eefxpot=create_EEFxPot("eefxpot",
                    selPairs=[("segid A","segid A")] +
                    [("not name K and segid A",f"segid {segid}") for segid in
                     "B C D".split()])
eefxpot.setVerbose(False)
potList.append(eefxpot)
rampedParams.append(MultRamp(0.1,1.0,"eefxpot.setScale(VALUE)"))
rampedParams.append(StaticRamp("repel.setScale(0)"))

# setup parameters for atom-atom repulsive term. (van der Waals-like term)
#
#from repelPotTools import create_RepelPot,initRepel
#repel = create_RepelPot('repel')
#potList.append(repel)
#rampedParams.append( StaticRamp("initRepel(repel,use14=False)") )
#rampedParams.append( MultRamp(.004,4,  "repel.setScale( VALUE)") )

#use repel at high temp
#
# setup parameters for atom-atom repulsive term. (van der Waals-like term)
#
```

```
from repelPotTools import create_RepelPot,initRepel
repel = create_RepelPot('repel',
                    selPairs=[("segid A","segid A")] +
                    [("not name K and segid A",f"segid {segid}") for segid in
                     "B C D".split()])

potList.append(repel)
#rampedParams.append( StaticRamp("initRepel(repel,use14=False)") )
#rampedParams.append( MultRamp(.004,4,  "repel.setScale( VALUE)") )
# nonbonded interaction only between CA atoms
highTempParams.append( StaticRamp("""initRepel(repel,
                                        use14=True,
                                        scale=0.004,
                                        repel=1.2,
                                        moveTol=45,
                                        interactingAtoms='name CA'
                                        )""") )
hiTempParams.append(StaticRamp("repel.setScale(1)"))
hiTempParams.append(StaticRamp("eefxpot.setScale(0)"))

# a term to keep the centroids of subunits A and C the same spread
# as the starting structure
from posDiffPotTools import create_PosDiffPot
acSpread = create_PosDiffPot("acSpread",
                         "segid A C and name CA and resid 1:333",
```

```
                        cmpSel=None,
                        pdbFile=startPDB)
#for range in " 36:100 101:200 201:301".split():
#    acSpread.addEquivAtomSelPair(f"segid A and resid {range} and name CA",
#                                 f"segid C and resid {range} and name CA")
#    pass
acSpread.setUpperBound(1)
potList.append( acSpread )
#from posDiffPotTools import create_PosDiffPot
#bdSpread = create_PosDiffPot("bdSpread",
#                        #selection = "segid A C and name CA and resid 36:301" ,
#                        selection = "segid A C and name O and resid 110:114",
#                        cmpSel=None,
#                        pdbFile=startPDB)
#for range in " 36:100 101:200 201:301".split():
#    acSpread.addEquivAtomSelPair(f"segid A and resid {range} and name CA",
#                                 f"segid C and resid {range} and name CA")
  # pass
#bdSpread.setUpperBound(0)
#potList.append( bdSpread )
#from psolPotTools import create_PSolPot
#psol = create_PSolPot("psol",
#file='KB11_PConly_4msHHmix_50msCCmix_7blocks_water_edited_deconvoluted.tbl')
#psol.setRmin(0.1)
#psol.setThreshold(0)
#psol.setProbeRadius( 4.0 )
```

```
#psol.setTargetType("correlation")
#potList.append(psol)
#
#psol.setFixTauc(True)
#psol.setTauC( 0.2 )
#psol.setSqn(3.5)      # electron spin quantum number
#psol.setRho0( 4 )
#psol.tessellation().setMoveTol(0.7)
#psol.setScale(1000)


simulation.makeCurrent( xplor.simulation )


# Set up dihedral angles
from xplorPot import XplorPot
torsionFile="POPC_1_to_301_xplor_restraint.tbl"
protocol.initDihedrals(torsionFile,
                useDefaults=False  # by default, symmetric sidechain
                          # restraints are included
                )
potList.append( XplorPot('CDIH') )
highTempParams.append( StaticRamp("potList['CDIH'].setScale(10)") )
rampedParams.append( StaticRamp("potList['CDIH'].setScale(200)") )
# set custom values of threshold values for violation calculation
#
potList['CDIH'].setThreshold( 5 ) #5 degrees is the default value, though
```

```
#
## hbda - distance/angle bb hbond term
##
#protocol.initHBDA('hbda.tbl')
#potList.append( XplorPot('HBDA') )

#New torsion angle database potential
#
from torsionDBPotTools import create_TorsionDBPot
torsionDB = create_TorsionDBPot('torsionDB', system='protein')
potList.append( torsionDB )
rampedParams.append( MultRamp(.002,2,"torsionDB.setScale(VALUE)") )#first differences.

#
# Statistical torsion angle potential
#
from torsionDBPotTools import create_TorsionDBPot
torsionDBPot = create_TorsionDBPot('tDB')
potList.append( torsionDBPot )
rampedParams.append( MultRamp(.002,2,"torsionDBPot.setScale(VALUE)") )


#covalent terms
from xplorPot import XplorPot
for term in ('BOND', 'ANGL', 'IMPR'):
```

```
    potList.append( XplorPot(term) )
    pass

# Set threshold for terms in potList to allow violation analysis.
potList['ANGL'].setThreshold(5.0)   # default is 2.0
potList['IMPR'].setThreshold(5.0)   # default is 2.0
# Use default values for the rest (bond: 0.05, cdih: 5.0, noe: 0.5).

rampedParams.append(MultRamp(0.4, 1.0, "potList['ANGL'].setScale(VALUE)"))
rampedParams.append(MultRamp(0.1, 1.0, "potList['IMPR'].setScale(VALUE)"))

from simulationTools import analyze
print( analyze(potList) )



# Give atoms uniform weights, except for the anisotropy axis
#
protocol.massSetup()



# IVM setup
#   the IVM is used for performing dynamics and minimization in torsion-angle
#   space, and in Cartesian space.
#
```

```
# IVM object used to randomize N-terminal tail
from ivm import IVM
randomizeIVM = IVM()
randomizeIVM.group("resid 36:301")

protocol.torsionTopology(randomizeIVM)



from ivm import IVM
dyn = IVM()
#dyn.fix(sele_group)

protocol.torsionTopology(dyn)

# minc used for final cartesian minimization
#
minc = IVM()
protocol.initMinimize(minc)
#minc.fix(sele_group)
protocol.cartesianTopology(minc)
#minc.fix("resid 110:116 and name CA N O C")



# object which performs simulated annealing
#
```

```
from simulationTools import AnnealIVM

temp_ini = 3500.0

temp_fin = 25.0


#def accept(potList):
#    """
#    return True if current structure meets acceptance criteria
#    """
#    if potList['noe'].violations()>0:
#        return False
#    if potList['rdc'].rms()>1.2: #this might be tightened some
#        return False
#    if potList['CDIH'].violations()>0:
#        return False
#    if potList['BOND'].violations()>0:
#        return False
#    if potList['ANGL'].violations()>0:
#        return False
#    if potList['IMPR'].violations()>1:
#        return False
#
#    return True
#
def calcOneStructure(loopInfo):
    """ this function calculates a single structure, performs analysis on the
```

```
structure, and then writes out a pdb file, with remarks.
"""


# Generate an initial structure by randomizing torsion angles.
from monteCarlo import randomizeTorsions
randomizeTorsions(randomizeIVM)
protocol.fixupCovalentGeom(maxIters=100, useVDW=1)


#
# High Temperature Dynamics Stage.
#
# Set torsion angles from restraints.
# (They start satisfied, allowing the shortening of high temp dynamics.)
from torsionTools import setTorsionsFromTable
setTorsionsFromTable(torsionFile)


# Initialize parameters for high temp dynamics.
from simulationTools import InitialParams
InitialParams(rampedParams) # overides some in rampedParams
InitialParams(hiTempParams) # overides some in rampedParams


#
# Torsion angle minimization.
#
protocol.initMinimize(dyn,
                      potList=[term for term in potList
```

```
                    if term.instanceName() not in
                    "eefxpot psol".split()],
                numSteps=500,
                printInterval=10)
dyn.run()
from simulationTools import analyze




#dynamics with all-atom repel potential
protocol.initDynamics(dyn,
                potList=[term for term in potList
                    if term.instanceName()!="eefxpot"],
                bathTemp=temp_ini,
                finalTime=40,  # run for finalTime or
                        # numSteps * 0.001, whichever is less
                numSteps=800 if quick else 800,
                printInterval=10)


dyn.setETolerance(temp_ini/100) # used to det. stepsize. default: temp/1000
dyn.run()

InitialParams(rampedParams)
```

```
#minmization with EEFx potential
protocol.initMinimize(dyn,
                      potList=potList,
                      numSteps=200,
                      printInterval=10)
dyn.run()

#dynamics with EEFx potential
protocol.initDynamics(dyn,
                      potList=[term for term in potList
                               if term.instanceName()!="repel"],
                      bathTemp=temp_ini,
                      initVelocities=1,
                      finalTime=30,  # run for finalTime or
                                     # numSteps * 0.001, whichever is less
                      numSteps=300 if quick else 30000,
                      printInterval=100)

dyn.setETolerance(temp_ini/100) # used to det. stepsize. default: temp/1000
dyn.run()



#
# Simulated Annealing Stage.
#
```

```
# Initialize parameters for annealing.
InitialParams(rampedParams)

# Set up IVM object for annealing.
protocol.initDynamics(dyn,
                    potList=[term for term in potList
                            if term.instanceName()!="repel"],
                    finalTime=0.4,  # run for finalTime or
                                # numSteps * 0.001, whichever is less
                    numSteps=20 if quick else 201,
                    printInterval=100)

# Set up cooling loop and run.
from simulationTools import AnnealIVM
AnnealIVM(initTemp=temp_ini,
          finalTemp=temp_fin,
          tempStep=100 if quick else 12.5,
          ivm=dyn,
          rampedParams=rampedParams).run()

#
# Torsion angle minimization.
#
protocol.initMinimize(dyn,
                    potList=[term for term in potList
```

```
                        if term.instanceName()!="repel"],
                printInterval=50)
    if not quick: dyn.run()


    #
    # Final Cartesian minimization.
    #
    protocol.initMinimize(minc,
                potList=[term for term in potList
                        if term.instanceName()!="repel"],
                dEPred=10)
    if not quick: minc.run()


    #do analysis and write structure when this function returns
    protocol.writePDB(loopInfo.filename()+".full",
              selection=AtomSel("all",symSim))
    pass



from simulationTools import FinalParams
from simulationTools import StructureLoop
StructureLoop(numStructures=numberOfStructures,
          doWriteStructures=True,  #analyze and write coords after calc
          averagePotList=potList,
          averageContext=FinalParams(rampedParams),
```

```
        #calcMissingStructs=True, #calculate only missing structures
        pdbTemplate="SCRIPT_STRUCTURE.sa",
        structLoopAction=calcOneStructure,
        genViolationStats=True,
        averageTopFraction=0.2, #report only on best 20% of structs
#         averageAccept=accept,   #only use structures which pass accept()
        averageFilename="SCRIPT_ave.pdb",    #generate regularized ave structure
        averageCompSel="not resname ANI and not name H*"     ).run()
```

Python script for Xplor-NIH for second step of refinement with EEFx

```
import protocol
import prePot
#xplor.requireVersion("2.4")

#
# this version refines from a reasonable model structure.
#
# CDS 2005/05/10
#

(opts,args) = xplor.parseArguments(["quick"]) # check for command-line typos

quick=False
for opt in opts:
    if opt[0]=="quick":  #specify -quick to just test that the script runs
        quick=True
        pass
    pass

numberOfStructures=10

if quick:
    numberOfStructures=1
```

```
    pass


# protocol module has many high-level helper functions.
#
import protocol
protocol.initRandomSeed(3421)   #explicitly set random seed


# INITIALIZE PARAMETERS AND TOPOLOGY.
import eefxPotTools
eefxPotTools.initEEFx()


#
# annealing settings
#


command = xplor.command


protocol.initParams("protein")


#
#protocol.genExtendedStructure("gb1_extended_%d.pdb" %
#                              protocol.initialRandomSeed())


import protocol
```

```
startPDB='Rosetta_tetramer_full_length_I131C_I131C.pdb'
protocol.loadPDB(startPDB,deleteUnknownAtoms=True)

xplor.simulation.deleteAtoms("segid B C D")


from atomSelAction import SetProperty
AtomSel("all").apply( SetProperty("segmentName","") )

import symSimSetup
symSim = symSimSetup.init()
######################################################################################
##
import glob
protocol.initCoords(glob.glob("test_38_EEFX_14.sa")[0])
#protocol.initCoords(erase=True,
                    #selection="resid 198 199 209 and not name C CA N O")
protocol.addUnknownAtoms()
######################################################################################
##
##generate distance restraints from the starting structure
#if xplor.p_processID==0:
#    from noePotTools import genRestraints
#    open('distance_K.tbl','w').write(genRestraints("name O K and resid 110:116 402:404 ",
#                                        distCutoff=6.,
#                                        upperBound=2.0,
```

```
#                                        lowerBound=1.0)
#                              )
#    pass

#
# a PotList contains a list of potential terms. This is used to specify which
# terms are active during refinement.
#
from potList import PotList
potList = PotList()
crossTerms = PotList('cross')

# parameters to ramp up during the simulated annealing protocol
#
from simulationTools import MultRamp, StaticRamp, InitialParams

rampedParams=[]
highTempParams=[]

# compare atomic Cartesian rmsd with a reference structure
#  backbone and heavy atom RMSDs will be printed in the output
#  structure files
#
from posDiffPotTools import create_PosDiffPot
protomerNCS = create_PosDiffPot("ncs",
```

```
                    "name CA C O N and resid 46:150 161:281",
                    selection2="segid A and name CA C O N and resid 46:150 161:281",
                    pdbFile=startPDB)

protomerNCS.setScale( 5 )
protomerNCS.setUpperBound( 0 )
potList.append( protomerNCS )
##allowed to be up to 3 Angstroms from starting structure at the end of the
## calculation
#rampedParams.append( MultRamp(1,3, "protomerNCS.setUpperBound( VALUE )") )



import simulation
simulation.makeCurrent( symSim )

## gyration volume term
##
#from gyrPotTools import create_GyrPot
#gyr = create_GyrPot("Vgyr",
#                   "resid 36:300") # selection should exclude disordered tails
#potList.append(gyr)
#rampedParams.append( MultRamp(.002,1,"gyr.setScale(VALUE)") )

# set up NOE potential
noe=PotList('noe')
potList.append(noe)
```

```
from noePotTools import create_NOEPot
for (name,scale,file) in [('all',1,"distance_K.tbl"),
                          #add entries for additional tables
                          ]:
    pot = create_NOEPot(name,file)
    pot.setNMono(4)
#    # pot.setPotType("soft") # if you think there may be bad NOEs#
    pot.setScale(scale)
    noe.append(pot)
rampedParams.append( MultRamp(2,30, "noe.setScale( VALUE )") )


from noePotTools import create_NOEPot
IntraDist = create_NOEPot ("IntraDist","KB11_POPC_Intra_distances.tbl")
potList.append(IntraDist)
rampedParams.append( MultRamp(2,30, "IntraDist.setScale( VALUE )") )


from noePotTools import create_NOEPot
InterDist = create_NOEPot("InterDist","KB11_POPC_Inter_distances.tbl")
potList.append(InterDist)
rampedParams.append( MultRamp(2,30, "InterDist.setScale( 4*VALUE )"))
# HBPot - knowledge-based hydrogen bond term
#
from hbPotTools import create_HBPot
hb = create_HBPot('hb')
hb.setScale(2.5)
```

```
potList.append( hb )

#nonbonded term - EEFx
from eefxPotTools import create_EEFxPot, param_LK
eefxpot=create_EEFxPot("eefxpot",
                    selPairs=[("segid A","segid A")] +
                    [("not name K and segid A",f"segid {segid}") for segid in
                     "B C D".split()])
eefxpot.setVerbose(False)
potList.append(eefxpot)
rampedParams.append(MultRamp(0.1,1.0,"eefxpot.setScale(VALUE)"))
rampedParams.append(StaticRamp("repel.setScale(0)"))

# setup parameters for atom-atom repulsive term. (van der Waals-like term)
#
#from repelPotTools import create_RepelPot,initRepel
#repel = create_RepelPot('repel')
#potList.append(repel)
#rampedParams.append( StaticRamp("initRepel(repel,use14=False)") )
#rampedParams.append( MultRamp(.004,4,  "repel.setScale( VALUE)") )

#use repel at high temp
#
# setup parameters for atom-atom repulsive term. (van der Waals-like term)

from repelPotTools import create_RepelPot,initRepel
```

```
repel = create_RepelPot('repel',
                 selPairs=[("segid A","segid A")] +
                 [("segid A",
                   f"not name K* and segid {segid}") for segid in
                  "B C D".split()])


potList.append(repel)
rampedParams.append( StaticRamp("initRepel(repel,use14=False)") )
rampedParams.append( MultRamp(.004,4,  "repel.setScale( VALUE)") )
# nonbonded interaction only between CA atoms
highTempParams.append( StaticRamp("""initRepel(repel,
                                    use14=True,
                                    scale=0.004,
                                    repel=1.2,
                                    moveTol=45,
                                    interactingAtoms='name CA'
                                    )""") )
highTempParams.append(StaticRamp("repel.setScale(1)"))
highTempParams.append(StaticRamp("eefxpot.setScale(0)"))


# a term to keep the centroids of subunits A and C the same spread
# as the starting structure
from posDiffPotTools import create_PosDiffPot
acSpread = create_PosDiffPot("acSpread",
                       "segid A C and name CA and resid 1:333",
```

```
                    cmpSel=None,
                    pdbFile=startPDB)
#for range in " 36:100 101:200 201:301".split():
#    acSpread.addEquivAtomSelPair(f"segid A and resid {range} and name CA",
#                                 f"segid C and resid {range} and name CA")
#    pass
acSpread.setUpperBound(1)
potList.append( acSpread )

from psolPotTools import create_PSolPot
psol = create_PSolPot("psol",domainSelection="not (PSEUDO or name K)",
file='KB11_PConly_4msHHmix_50msCCmix_7blocks_water_edited_deconvoluted.tbl')
psol.setRmin(0.1)
psol.setThreshold(0)
psol.setProbeRadius( 4.0 )
psol.setTargetType("correlation")
potList.append(psol)
#
psol.setFixTauc(True)
psol.setTauC( 0.2 )
psol.setSqn(3.5)      # electron spin quantum number
psol.setRho0( 4 )
psol.tessellation().setMoveTol(0.7)
psol.setScale(1000)

simulation.makeCurrent( xplor.simulation )
```

```
# Set up dihedral angles
from xplorPot import XplorPot
torsionFile="POPC_1_to_301_xplor_restraint.tbl"
protocol.initDihedrals(torsionFile,
                   useDefaults=False  # by default, symmetric sidechain
                                      # restraints are included
                   )
potList.append( XplorPot('CDIH') )
highTempParams.append( StaticRamp("potList['CDIH'].setScale(10)") )
rampedParams.append( StaticRamp("potList['CDIH'].setScale(200)") )
# set custom values of threshold values for violation calculation
#
potList['CDIH'].setThreshold( 5 ) #5 degrees is the default value, though


#
## hbda - distance/angle bb hbond term
##
#protocol.initHBDA('hbda.tbl')
#potList.append( XplorPot('HBDA') )


#New torsion angle database potential
#
from torsionDBPotTools import create_TorsionDBPot
torsionDB = create_TorsionDBPot('torsionDB', system='protein')
```

```
potList.append( torsionDB )
rampedParams.append( MultRamp(.002,2,"torsionDB.setScale(VALUE)") )#first differences.


#
# Statistical torsion angle potential
#
from torsionDBPotTools import create_TorsionDBPot
torsionDBPot = create_TorsionDBPot('tDB')
potList.append( torsionDBPot )
rampedParams.append( MultRamp(.002,2,"torsionDBPot.setScale(VALUE)") )



#covalent terms
from xplorPot import XplorPot
for term in ('BOND', 'ANGL', 'IMPR'):
   potList.append( XplorPot(term) )
   pass

# Set threshold for terms in potList to allow violation analysis.
potList['ANGL'].setThreshold(5.0)   # default is 2.0
potList['IMPR'].setThreshold(5.0)   # default is 2.0
# Use default values for the rest (bond: 0.05, cdih: 5.0, noe: 0.5).

rampedParams.append(MultRamp(0.4, 1.0, "potList['ANGL'].setScale(VALUE)"))
rampedParams.append(MultRamp(0.1, 1.0, "potList['IMPR'].setScale(VALUE)"))
```

```
from simulationTools import analyze
print( analyze(potList) )



# Give atoms uniform weights, except for the anisotropy axis
#
protocol.massSetup()



# IVM setup
#   the IVM is used for performing dynamics and minimization in torsion-angle
#   space, and in Cartesian space.
#

# IVM object used to randomize N-terminal tail
from ivm import IVM
randomizeIVM = IVM()
randomizeIVM.group("resid 36:301 and name C CA N")

protocol.torsionTopology(randomizeIVM)



from ivm import IVM
dyn = IVM()
```

```
protocol.torsionTopology(dyn)

# minc used for final cartesian minimization
#
minc = IVM()
protocol.initMinimize(minc)

protocol.cartesianTopology(minc)
#minc.fix("resid 110:116 and name CA N O C")



# object which performs simulated annealing
#
from simulationTools import AnnealIVM
temp_ini = 3500.0
temp_fin = 25.0

#def accept(potList):
#    """
#    return True if current structure meets acceptance criteria
#    """
#    if potList['noe'].violations()>0:
#        return False
#    if potList['rdc'].rms()>1.2: #this might be tightened some
```

```
#        return False
#    if potList['CDIH'].violations()>0:
#        return False
#    if potList['BOND'].violations()>0:
#        return False
#    if potList['ANGL'].violations()>0:
#        return False
#    if potList['IMPR'].violations()>1:
#        return False
#
#    return True
#
def calcOneStructure(loopInfo):
    """ this function calculates a single structure, performs analysis on the
    structure, and then writes out a pdb file, with remarks.
    """

    # Generate an initial structure by randomizing torsion angles.
    #from monteCarlo import randomizeTorsions
    #randomizeTorsions(randomizeIVM)
    #protocol.fixupCovalentGeom(maxIters=100, useVDW=1)

    #
    # High Temperature Dynamics Stage.
    #
```

```
# Set torsion angles from restraints.
# (They start satisfied, allowing the shortening of high temp dynamics.)
#from torsionTools import setTorsionsFromTable
#setTorsionsFromTable(torsionFile)

# Initialize parameters for high temp dynamics.
from simulationTools import InitialParams
InitialParams(rampedParams) # overides some in rampedParams
InitialParams(highTempParams) # overides some in rampedParams

#minimization with all-atom repel potential
protocol.initMinimize(dyn,
                potList=[term for term in potList
                    if term.instanceName() not in
                    "eefxpot psol noe".split()],
                numSteps=800 if quick else 800,
                printInterval=10)


dyn.run()
#minimization with all-atom repel potential
protocol.initMinimize(dyn,
                potList=[term for term in potList
                    if term.instanceName() not in
                    "eefxpot psol".split()],
                numSteps=800 if quick else 800,
```

```
                printInterval=10)



dyn.run()

protocol.writePDB("t.pdb")
print( analyze(potList) )
#exit()
#
# Torsion angle minimization.
#
protocol.initDynamics(dyn,
                potList=[term for term in potList
                      if term.instanceName() not in
                      "eefxpot psol".split()],
                bathTemp=temp_ini,
                initVelocities=1,
                finalTime=30,  # run for finalTime or
                          # numSteps * 0.001, whichever is less
                numSteps=300 if quick else 30000,
                printInterval=100)

dyn.setETolerance(temp_ini/100) # used to det. stepsize. default: temp/1000
dyn.run()
```

```
#minimization with all-atom repel potential
protocol.initMinimize(dyn,
                potList=[term for term in potList
                       if term.instanceName() not in
                       "eefxpot psol".split()],
                numSteps=800 if quick else 800,
                printInterval=10)



dyn.run()


InitialParams(rampedParams)


#minmization with EEFx potential
protocol.initMinimize(dyn,
                potList=potList,
                numSteps=200,
                printInterval=10)
dyn.run()


#dynamics with EEFx potential
protocol.initDynamics(dyn,
                potList=[term for term in potList
```

```
          if term.instanceName() not in
          "eefxpot".split()],
      bathTemp=temp_ini,
      initVelocities=1,
      finalTime=30,  # run for finalTime or
            # numSteps * 0.001, whichever is less
      numSteps=300 if quick else 30000,
      printInterval=100)

dyn.setETolerance(temp_ini/100) # used to det. stepsize. default: temp/1000
dyn.run()



#
# Simulated Annealing Stage.
#
# Initialize parameters for annealing.
InitialParams(rampedParams)

# Set up IVM object for annealing.
protocol.initDynamics(dyn,
      potList=[term for term in potList
          if term.instanceName() not in
          "eefxpot".split()],
      finalTime=0.4,  # run for finalTime or
```

```
                    # numSteps * 0.001, whichever is less
                numSteps=20 if quick else 201,
                printInterval=100)

# Set up cooling loop and run.
from simulationTools import AnnealIVM
AnnealIVM(initTemp=temp_ini,
    finalTemp=temp_fin,
    tempStep=100 if quick else 12.5,
    ivm=dyn,
    rampedParams=rampedParams).run()

#
# Torsion angle minimization.
#
protocol.initMinimize(dyn,
                potList=[term for term in potList
                    if term.instanceName()!="eefxpot"],
                printInterval=50)
if not quick: dyn.run()

#
# Final Cartesian minimization.
#
protocol.initMinimize(minc,
                potList=[term for term in potList
```

```
                    if term.instanceName()!="eefxpot"],
                dEPred=10)
    if not quick: minc.run()

    #do analysis and write structure when this function returns
    protocol.writePDB(loopInfo.filename()+".full",
                selection=AtomSel("all",symSim))
    pass




from simulationTools import FinalParams
from simulationTools import StructureLoop
StructureLoop(numStructures=numberOfStructures,
        doWriteStructures=True,  #analyze and write coords after calc
        averageFitSel=None,
        averagePotList=potList,
        averageContext=FinalParams(rampedParams),
        #calcMissingStructs=True, #calculate only missing structures
        pdbTemplate="SCRIPT_STRUCTURE.sa",
        structLoopAction=calcOneStructure,
        genViolationStats=True,
        averageTopFraction=0.2, #report only on best 20% of structs
#       averageAccept=accept,   #only use structures which pass accept()
        averageFilename="SCRIPT_ave.pdb",    #generate regularized ave structure
```

```
averageCompSel="not resname ANI and not name H*"     ).run()
```

Python script for Xplor-NIH for first step of refinement with IMMx

```
import protocol
#import prePot
#xplor.requireVersion("2.4")


#
# this version refines from a reasonable model structure.
#
# CDS 2005/05/10
#



(opts,args) = xplor.parseArguments(["quick"]) # check for command-line typos


quick=False
for opt in opts:
   if opt[0]=="quick":  #specify -quick to just test that the script runs
      quick=True
      pass
   pass



numberOfStructures=100


if quick:
```

```
    numberOfStructures=1
    pass

# protocol module has many high-level helper functions.
#
import protocol
protocol.initRandomSeed(3421)   #explicitly set random seed

# INITIALIZE PARAMETERS AND TOPOLOGY.
import eefxPotTools
eefxPotTools.initEEFx()


#
# annealing settings
#
#sele_group = "resid 108:116"
command = xplor.command

protocol.initParams("protein")


#
#protocol.genExtendedStructure("gb1_extended_%d.pdb" %
#                             protocol.initialRandomSeed())

import protocol
```

```
startPDB='Rosetta_tetramer_4k_I131C.pdb'
protocol.loadPDB(startPDB,deleteUnknownAtoms=True)

xplor.simulation.deleteAtoms("segid B C D")

from atomSelAction import SetProperty
AtomSel("all").apply( SetProperty("segmentName","") )

import symSimSetup
symSim = symSimSetup.init()
##generate distance restraints from the starting structure
if xplor.p_processID==0:
   from noePotTools import genRestraints
   open('distance_K.tbl','w').write(genRestraints("name O K and resid 108:114 401:404 ",
                                                  distCutoff=20.0,
                                                  upperBound=0.0,
                                                  lowerBound=0.0)
                                    )
   pass

#
# a PotList contains a list of potential terms. This is used to specify which
# terms are active during refinement.
#
from potList import PotList
```

```
potList = PotList()
crossTerms = PotList('cross')


# parameters to ramp up during the simulated annealing protocol
#
from simulationTools import MultRamp, StaticRamp, InitialParams


rampedParams=[]
highTempParams=[]


# compare atomic Cartesian rmsd with a reference structure
#  backbone and heavy atom RMSDs will be printed in the output
#  structure files
#
from posDiffPotTools import create_PosDiffPot
protomerNCS = create_PosDiffPot("ncs",
                          "name CA C O N and resid 46:150 161:281",
                          selection2="segid A and name CA C O N and resid 46:150 161:281",
                          pdbFile=startPDB)


protomerNCS.setScale( 5 )
protomerNCS.setUpperBound( 0 )
potList.append( protomerNCS )
##allowed to be up to 3 Angstroms from starting structure at the end of the
## calculation
#rampedParams.append( MultRamp(1,3, "protomerNCS.setUpperBound( VALUE )") )
```

```
# lists to hold force constant settings for various stages of
# structure calculation
from simulationTools import MultRamp, StaticRamp
hiTempParams = []
# Settings for annealing stage.
rampedParams = []

# set up NOE potential
noe=PotList('noe')
potList.append(noe)
from noePotTools import create_NOEPot
for (name,scale,file) in [('all',1,"KB11_POPC_Intra_distances.tbl"),
                          #add entries for additional tables
                          ]:
    pot = create_NOEPot(name,file)
    # pot.setPotType("soft") # if you think there may be bad NOEs
    pot.setScale(scale)
    noe.append(pot)
rampedParams.append( MultRamp(2,30, "noe.setScale( VALUE )") )


from noePotTools import create_NOEPot
Kdist = create_NOEPot("K-dist","distance_K.tbl")
potList.append(Kdist)
```

```
rampedParams.append( MultRamp(2,30, "Kdist.setScale( 10*VALUE )") )


import simulation
simulation.makeCurrent( symSim )


## gyration volume term
##
#from gyrPotTools import create_GyrPot
#gyr = create_GyrPot("Vgyr",
#                    "resid 36:300") # selection should exclude disordered tails
#potList.append(gyr)
#rampedParams.append( MultRamp(.002,1,"gyr.setScale(VALUE)") )
# set up NOE potential
restraints=""
for resid in range(401,404):
   kAtoms= AtomSel(f"resid {resid} and name K*")
   from itertools import combinations
   for atom1,atom2 in combinations(kAtoms,2):
      restraints += f"assi (atom {atom1.segmentName()} {resid} K)\n"
      restraints += f"assi (atom {atom2.segmentName()} {resid} K) "
      restraints += " 2 2 0\n"
      pass
   pass
print( restraints)
Kaxis = create_NOEPot("K-axis",restraints=restraints)
potList.append(Kaxis)
```

```
rampedParams.append( MultRamp(2,30, "Kdist.setScale( VALUE )") )


from noePotTools import create_NOEPot
InterDist = create_NOEPot("Inter-Dist","KB11_POPC_Inter_distances.tbl")
potList.append(InterDist)
rampedParams.append( MultRamp(2,30, "InterDist.setScale( 4*VALUE )") )


# HBPot - knowledge-based hydrogen bond term
#
from hbPotTools import create_HBPot
hb = create_HBPot('hb')
hb.setScale(2.5)
potList.append( hb )


#nonbonded term - EEFx
from eefxPotTools import create_EEFxPot, param_LK
eefxpot=create_EEFxPot("eefxpot",
                    selPairs=[("segid A","segid A")] +
                    [("not name K and segid A",f"segid {segid}") for segid in
                     "B C D".split()])
eefxpot.setVerbose(False)
##########################################################################################
######################
eefxpot.setScale(1)
# eefxpot.setVerbose(1)
eefxpot.setIMMx(1)
```

eefxpot.setMoveTol(0.5)

print(eefxpot.showParam())

eefxpot.setThickness(27) # IMMx membrane thickness [25.4 DMPC; 28.6 DPPC; 27.0 POPC; 29.6 DOPC].

eefxpot.setProfileN(2) # IMMx n parameter of membrane profile (use n<3 in early stages).

eefxpot.setA(0.85)   # IMMx a value that scales dielectric screening. Dflt=[0.85].

IMM_com = "segid A B C D and resid 46:150 and (name CA)"   # Center of mass selection for IMMx position.

Zpos=0   # Z position relative to IMMx membrane center. Dflt=[0].

from eefxPotTools import setCenter, setCenterXY

setCenter(IMM_com, Zpos)   # Translate selected center of mass to IMMx Zpos.

###

potList.append(eefxpot)

rampedParams.append(MultRamp(0.1,1.0,"eefxpot.setScale(VALUE)"))

rampedParams.append(StaticRamp("repel.setScale(0)"))

setup parameters for atom-atom repulsive term. (van der Waals-like term)

#

#from repelPotTools import create_RepelPot,initRepel

#repel = create_RepelPot('repel')

#potList.append(repel)

#rampedParams.append(StaticRamp("initRepel(repel,use14=False)"))

#rampedParams.append(MultRamp(.004,4, "repel.setScale(VALUE)"))

#use repel at high temp

```
#
# setup parameters for atom-atom repulsive term. (van der Waals-like term)
#
from repelPotTools import create_RepelPot,initRepel
repel = create_RepelPot('repel',
                    selPairs=[("segid A","segid A")] +
                    [("not name K and segid A",f"segid {segid}") for segid in
                     "B C D".split()])

potList.append(repel)
#rampedParams.append( StaticRamp("initRepel(repel,use14=False)") )
#rampedParams.append( MultRamp(.004,4,  "repel.setScale( VALUE)") )
# nonbonded interaction only between CA atoms
highTempParams.append( StaticRamp("""initRepel(repel,
                                        use14=True,
                                        scale=0.004,
                                        repel=1.2,
                                        moveTol=45,
                                        interactingAtoms='name CA'
                                        )""") )
hiTempParams.append(StaticRamp("repel.setScale(1)"))
hiTempParams.append(StaticRamp("eefxpot.setScale(0)"))

# a term to keep the centroids of subunits A and C the same spread
# as the starting structure
```

```
from posDiffPotTools import create_PosDiffPot
acSpread = create_PosDiffPot("acSpread",
                             "segid A C and name CA and resid 1:333",
                             cmpSel=None,
                             pdbFile=startPDB)
#for range in " 36:100 101:200 201:301".split():
#    acSpread.addEquivAtomSelPair(f"segid A and resid {range} and name CA",
#                                 f"segid C and resid {range} and name CA")
#    pass
acSpread.setUpperBound(1)
potList.append( acSpread )

#from posDiffPotTools import create_PosDiffPot
#bdSpread = create_PosDiffPot("bdSpread",
                             #selection = "segid A C and name CA and resid 36:301" ,
#                              selection = "segid B D and name O and resid 110:114",
#                              cmpSel=None,
#                              pdbFile=startPDB)
#for range in " 36:100 101:200 201:301".split():
#    acSpread.addEquivAtomSelPair(f"segid A and resid {range} and name CA",
#                                 f"segid C and resid {range} and name CA")
  # pass
#bdSpread.setUpperBound(1)
#potList.append( bdSpread )

#from posDiffPotTools import create_PosDiffPot
```

```
#kkSpread = create_PosDiffPot("kkSpread",
#                              #selection = "segid A C and name CA and resid 36:301" ,
#                              selection = "segid A A and name K and resid 401:404",
#                              cmpSel=None,
#                              pdbFile=startPDB)
##for range in " 36:100 101:200 201:301".split():
#    acSpread.addEquivAtomSelPair(f"segid A and resid {range} and name CA",
#                                  f"segid C and resid {range} and name CA")
  # pass
#kkSpread.setUpperBound(0)
#potList.append( kkSpread )
#from psolPotTools import create_PSolPot
#psol = create_PSolPot("psol",
#file='KB11_PConly_4msHHmix_50msCCmix_7blocks_water_edited_deconvoluted.tbl')
#psol.setRmin(0.1)
#psol.setThreshold(0)
#psol.setProbeRadius( 4.0 )
#psol.setTargetType("correlation")
#potList.append(psol)
#
#psol.setFixTauc(True)
#psol.setTauC( 0.2 )
#psol.setSqn(3.5)      # electron spin quantum number
#psol.setRho0( 4 )
#psol.tessellation().setMoveTol(0.7)
```

```
#psol.setScale(1000)


simulation.makeCurrent( xplor.simulation )


# Set up dihedral angles
from xplorPot import XplorPot
torsionFile="POPC_1_to_301_xplor_restraint.tbl"
protocol.initDihedrals(torsionFile,
                useDefaults=False  # by default, symmetric sidechain
                                   # restraints are included
                )
potList.append( XplorPot('CDIH') )
highTempParams.append( StaticRamp("potList['CDIH'].setScale(10)") )
rampedParams.append( StaticRamp("potList['CDIH'].setScale(200)") )
# set custom values of threshold values for violation calculation
#
potList['CDIH'].setThreshold( 5 ) #5 degrees is the default value, though


#
## hbda - distance/angle bb hbond term
##
#protocol.initHBDA('hbda.tbl')
#potList.append( XplorPot('HBDA') )


#New torsion angle database potential
#
```

```
from torsionDBPotTools import create_TorsionDBPot
torsionDB = create_TorsionDBPot('torsionDB', system='protein')
potList.append( torsionDB )
rampedParams.append( MultRamp(.002,2,"torsionDB.setScale(VALUE)") )#first differences.

#
# Statistical torsion angle potential
#
from torsionDBPotTools import create_TorsionDBPot
torsionDBPot = create_TorsionDBPot('tDB')
potList.append( torsionDBPot )
rampedParams.append( MultRamp(.002,2,"torsionDBPot.setScale(VALUE)") )


#covalent terms
from xplorPot import XplorPot
for term in ('BOND', 'ANGL', 'IMPR'):
    potList.append( XplorPot(term) )
    pass

# Set threshold for terms in potList to allow violation analysis.
potList['ANGL'].setThreshold(5.0)   # default is 2.0
potList['IMPR'].setThreshold(5.0)   # default is 2.0
# Use default values for the rest (bond: 0.05, cdih: 5.0, noe: 0.5).
```

```
rampedParams.append(MultRamp(0.4, 1.0, "potList['ANGL'].setScale(VALUE)"))
rampedParams.append(MultRamp(0.1, 1.0, "potList['IMPR'].setScale(VALUE)"))

from simulationTools import analyze
print( analyze(potList) )



# Give atoms uniform weights, except for the anisotropy axis
#
protocol.massSetup()


# IVM setup
#   the IVM is used for performing dynamics and minimization in torsion-angle
#   space, and in Cartesian space.
#

# IVM object used to randomize N-terminal tail
from ivm import IVM
randomizeIVM = IVM()
randomizeIVM.group("resid 36:301")

protocol.torsionTopology(randomizeIVM)
```

```
from ivm import IVM
dyn = IVM()
#dyn.group(sele_group)

protocol.torsionTopology(dyn)

# minc used for final cartesian minimization
#
minc = IVM()
protocol.initMinimize(minc)
#minc.fix(sele_group)
protocol.cartesianTopology(minc)
#minc.fix("resid 110:116 and name CA N O C")


# object which performs simulated annealing
#
from simulationTools import AnnealIVM
temp_ini = 3500.0
temp_fin = 25.0

#def accept(potList):
#    """
#    return True if current structure meets acceptance criteria
#    """
```

```
#    if potList['noe'].violations()>0:
#        return False
#    if potList['rdc'].rms()>1.2: #this might be tightened some
#        return False
#    if potList['CDIH'].violations()>0:
#        return False
#    if potList['BOND'].violations()>0:
#        return False
#    if potList['ANGL'].violations()>0:
#        return False
#    if potList['IMPR'].violations()>1:
#        return False
#
#    return True
#
def calcOneStructure(loopInfo):
    """ this function calculates a single structure, performs analysis on the
    structure, and then writes out a pdb file, with remarks.
    """

    # Generate an initial structure by randomizing torsion angles.
    from monteCarlo import randomizeTorsions
    randomizeTorsions(randomizeIVM)
    protocol.fixupCovalentGeom(maxIters=100, useVDW=1)

    #
```

```
# High Temperature Dynamics Stage.
#
# Set torsion angles from restraints.
# (They start satisfied, allowing the shortening of high temp dynamics.)
from torsionTools import setTorsionsFromTable
setTorsionsFromTable(torsionFile)

# Initialize parameters for high temp dynamics.
from simulationTools import InitialParams
InitialParams(rampedParams) # overides some in rampedParams
InitialParams(hiTempParams) # overides some in rampedParams

#
# Torsion angle minimization.
#
protocol.initMinimize(dyn,
                      potList=[term for term in potList
                               if term.instanceName() not in
                               "eefxpot psol".split()],
                      numSteps=500,
                      printInterval=10)
dyn.run()
from simulationTools import analyze
```

```
#dynamics with all-atom repel potential
protocol.initDynamics(dyn,
                potList=[term for term in potList
                      if term.instanceName()!="eefxpot"],
                bathTemp=temp_ini,
                finalTime=40,  # run for finalTime or
                          # numSteps * 0.001, whichever is less
                numSteps=800 if quick else 800,
                printInterval=10)


dyn.setETolerance(temp_ini/100) # used to det. stepsize. default: temp/1000
dyn.run()

InitialParams(rampedParams)

#minmization with EEFx potential
protocol.initMinimize(dyn,
                potList=potList,
                numSteps=200,
                printInterval=10)
dyn.run()

#dynamics with EEFx potential
```

```
protocol.initDynamics(dyn,
                      potList=[term for term in potList
                               if term.instanceName()!="repel"],
                      bathTemp=temp_ini,
                      initVelocities=1,
                      finalTime=30,  # run for finalTime or
                                     # numSteps * 0.001, whichever is less
                      numSteps=300 if quick else 30000,
                      printInterval=100)

dyn.setETolerance(temp_ini/100) # used to det. stepsize. default: temp/1000
dyn.run()



#
# Simulated Annealing Stage.
#
# Initialize parameters for annealing.
InitialParams(rampedParams)

# Set up IVM object for annealing.
protocol.initDynamics(dyn,
                      potList=[term for term in potList
                               if term.instanceName()!="repel"],
                      finalTime=0.4,  # run for finalTime or
```

```
                # numSteps * 0.001, whichever is less
            numSteps=20 if quick else 201,
            printInterval=100)

# Set up cooling loop and run.
from simulationTools import AnnealIVM
AnnealIVM(initTemp=temp_ini,
    finalTemp=temp_fin,
    tempStep=100 if quick else 12.5,
    ivm=dyn,
    rampedParams=rampedParams).run()

#
# Torsion angle minimization.
#
protocol.initMinimize(dyn,
            potList=[term for term in potList
                if term.instanceName()!="repel"],
            printInterval=50)
if not quick: dyn.run()

#
# Final Cartesian minimization.
#
protocol.initMinimize(minc,
            potList=[term for term in potList
```

```
                    if term.instanceName()!="repel"],
               dEPred=10)
    if not quick: minc.run()

    #do analysis and write structure when this function returns
    protocol.writePDB(loopInfo.filename()+".full",
              selection=AtomSel("all",symSim))
    pass



from simulationTools import FinalParams
from simulationTools import StructureLoop
StructureLoop(numStructures=numberOfStructures,
          doWriteStructures=True,  #analyze and write coords after calc
          averagePotList=potList,
          averageContext=FinalParams(rampedParams),
          #calcMissingStructs=True, #calculate only missing structures
          pdbTemplate="SCRIPT_STRUCTURE.sa",
          structLoopAction=calcOneStructure,
          genViolationStats=True,
          averageTopFraction=0.2, #report only on best 20% of structs
#           averageAccept=accept,   #only use structures which pass accept()
          averageFilename="SCRIPT_ave.pdb",    #generate regularized ave structure
          averageCompSel="not resname ANI and not name H*"     ).run()
```

Python script for Xplor-NIH for second step of refinement with IMMx

```
import protocol

import prePot

#xplor.requireVersion("2.4")


#

# this version refines from a reasonable model structure.

#

# CDS 2005/05/10

#



(opts,args) = xplor.parseArguments(["quick"]) # check for command-line typos


quick=False

for opt in opts:

   if opt[0]=="quick":  #specify -quick to just test that the script runs

      quick=True

      pass

   pass



numberOfStructures=100


if quick:

   numberOfStructures=1
```

```
    pass


# protocol module has many high-level helper functions.
#
import protocol
protocol.initRandomSeed(3421)   #explicitly set random seed


# INITIALIZE PARAMETERS AND TOPOLOGY.
import eefxPotTools
eefxPotTools.initEEFx()


#
# annealing settings
#


command = xplor.command


protocol.initParams("protein")


#
#protocol.genExtendedStructure("gb1_extended_%d.pdb" %
#                              protocol.initialRandomSeed())


import protocol
```

```
startPDB='Rosetta_tetramer_full_length_I131C.pdb'
protocol.loadPDB(startPDB,deleteUnknownAtoms=True)

xplor.simulation.deleteAtoms("segid B C D")



from atomSelAction import SetProperty
AtomSel("all").apply( SetProperty("segmentName","") )

import symSimSetup
symSim = symSimSetup.init()
##########################################################################################
##
import glob
protocol.initCoords(glob.glob("test_IMMX_16_54.sa")[0])
#protocol.initCoords(erase=True,
                    #selection="resid 198 199 209 and not name C CA N O")
protocol.addUnknownAtoms()
##########################################################################################
##
##generate distance restraints from the starting structure
#if xplor.p_processID==0:
#    from noePotTools import genRestraints
#    open('distance_K.tbl','w').write(genRestraints("name O K and resid 110:116 402:404 ",
#                                    distCutoff=6.,
#                                    upperBound=2.0,
```

```
#                                         lowerBound=1.0)
#                              )
#    pass

#
# a PotList contains a list of potential terms. This is used to specify which
# terms are active during refinement.
#
from potList import PotList
potList = PotList()
crossTerms = PotList('cross')

# parameters to ramp up during the simulated annealing protocol
#
from simulationTools import MultRamp, StaticRamp, InitialParams

rampedParams=[]
highTempParams=[]

# compare atomic Cartesian rmsd with a reference structure
#  backbone and heavy atom RMSDs will be printed in the output
#  structure files
#
from posDiffPotTools import create_PosDiffPot
protomerNCS = create_PosDiffPot("ncs",
```

```
                    "name CA C O N and resid 46:150 161:281",
                    selection2="segid A and name CA C O N and resid 46:150 161:281",
                    pdbFile=startPDB)

protomerNCS.setScale( 5 )
protomerNCS.setUpperBound( 0 )
potList.append( protomerNCS )
##allowed to be up to 3 Angstroms from starting structure at the end of the
## calculation
#rampedParams.append( MultRamp(1,3, "protomerNCS.setUpperBound( VALUE )") )



import simulation
simulation.makeCurrent( symSim )


## gyration volume term
##
#from gyrPotTools import create_GyrPot
#gyr = create_GyrPot("Vgyr",
#               "resid 36:300") # selection should exclude disordered tails
#potList.append(gyr)
#rampedParams.append( MultRamp(.002,1,"gyr.setScale(VALUE)") )


# set up NOE potential
noe=PotList('noe')
potList.append(noe)
```

```
from noePotTools import create_NOEPot
for (name,scale,file) in [('all',1,"distance_K.tbl"),
                    #add entries for additional tables
                    ]:
   pot = create_NOEPot(name,file)
   pot.setNMono(4)
#    # pot.setPotType("soft") # if you think there may be bad NOEs#
   pot.setScale(scale)
   noe.append(pot)
rampedParams.append( MultRamp(2,30, "noe.setScale( VALUE )") )


from noePotTools import create_NOEPot
IntraDist = create_NOEPot ("IntraDist","KB11_POPC_Intra_distances.tbl")
potList.append(IntraDist)
rampedParams.append( MultRamp(2,30, "IntraDist.setScale( VALUE )") )


from noePotTools import create_NOEPot
InterDist = create_NOEPot("InterDist","KB11_POPC_Inter_distances.tbl")
potList.append(InterDist)
rampedParams.append( MultRamp(2,30, "InterDist.setScale( 4*VALUE )"))
# HBPot - knowledge-based hydrogen bond term
#
from hbPotTools import create_HBPot
hb = create_HBPot('hb')
hb.setScale(2.5)
```

```
potList.append( hb )


#nonbonded term - EEFx

from eefxPotTools import create_EEFxPot, param_LK

eefxpot=create_EEFxPot("eefxpot",

                    selPairs=[("segid A","segid A")] +

                    [("not name K and segid A",f"segid {segid}") for segid in

                     "B C D".split()])

eefxpot.setVerbose(False)

################################################################################
#####################

eefxpot.setScale(1)

# eefxpot.setVerbose(1)

eefxpot.setIMMx(1)

# eefxpot.setMoveTol(0.5)

print(eefxpot.showParam())

eefxpot.setThickness(27) # IMMx membrane thickness [25.4 DMPC; 28.6 DPPC; 27.0 POPC; 29.6
DOPC].

eefxpot.setProfileN(2)  # IMMx n parameter of membrane profile (use n<3 in early stages).

eefxpot.setA(0.85)       # IMMx a value that scales dielectric screening. Dflt=[0.85].

IMM_com = "segid A B C D and resid 46:150 and (name CA)"   # Center of mass selection for
IMMx position.

Zpos=0                                                            # Z position relative to
IMMx membrane center. Dflt=[0].

from eefxPotTools import setCenter, setCenterXY

setCenter(IMM_com, Zpos)                          # Translate selected center of mass to IMMx
Zpos.

################################################################################
#####################
```

```
potList.append(eefxpot)
rampedParams.append(MultRamp(0.1,1.0,"eefxpot.setScale(VALUE)"))
rampedParams.append(StaticRamp("repel.setScale(0)"))

# setup parameters for atom-atom repulsive term. (van der Waals-like term)
#
#from repelPotTools import create_RepelPot,initRepel
#repel = create_RepelPot('repel')
#potList.append(repel)
#rampedParams.append( StaticRamp("initRepel(repel,use14=False)") )
#rampedParams.append( MultRamp(.004,4,  "repel.setScale( VALUE)") )

#use repel at high temp
#
# setup parameters for atom-atom repulsive term. (van der Waals-like term)

from repelPotTools import create_RepelPot,initRepel
repel = create_RepelPot('repel',
                        selPairs=[("segid A","segid A")] +
                        [("segid A",
                          f"not name K* and segid {segid}") for segid in
                         "B C D".split()])

potList.append(repel)
rampedParams.append( StaticRamp("initRepel(repel,use14=False)") )
```

```
rampedParams.append( MultRamp(.004,4,  "repel.setScale( VALUE)") )
# nonbonded interaction only between CA atoms
highTempParams.append( StaticRamp("""initRepel(repel,
                                   use14=True,
                                   scale=0.004,
                                   repel=1.2,
                                   moveTol=45,
                                   interactingAtoms='name CA'
                                   )""") )
highTempParams.append(StaticRamp("repel.setScale(1)"))
highTempParams.append(StaticRamp("eefxpot.setScale(0)"))


# a term to keep the centroids of subunits A and C the same spread
# as the starting structure
from posDiffPotTools import create_PosDiffPot
acSpread = create_PosDiffPot("acSpread",
                      "segid A C and name CA and resid 1:333",
                      cmpSel=None,
                      pdbFile=startPDB)
#for range in " 36:100 101:200 201:301".split():
#    acSpread.addEquivAtomSelPair(f"segid A and resid {range} and name CA",
#                                 f"segid C and resid {range} and name CA")
#    pass
acSpread.setUpperBound(1)
potList.append( acSpread )
```

```
from psolPotTools import create_PSolPot
psol = create_PSolPot("psol",domainSelection="not (PSEUDO or name K)",
file='KB11_PConly_4msHHmix_50msCCmix_7blocks_water_edited_deconvoluted.tbl')
psol.setRmin(0.1)
psol.setThreshold(0)
psol.setProbeRadius( 4.0 )
psol.setTargetType("correlation")
potList.append(psol)
#
psol.setFixTauc(True)
psol.setTauC( 0.2 )
psol.setSqn(3.5)      # electron spin quantum number
psol.setRho0( 4 )
psol.tessellation().setMoveTol(0.7)
psol.setScale(1000)

simulation.makeCurrent( xplor.simulation )

# Set up dihedral angles
from xplorPot import XplorPot
torsionFile="POPC_1_to_301_xplor_restraint.tbl"
protocol.initDihedrals(torsionFile,
                    useDefaults=False  # by default, symmetric sidechain
                                    # restraints are included
                    )
```

```
potList.append( XplorPot('CDIH') )
highTempParams.append( StaticRamp("potList['CDIH'].setScale(10)") )
rampedParams.append( StaticRamp("potList['CDIH'].setScale(200)") )
# set custom values of threshold values for violation calculation
#
potList['CDIH'].setThreshold( 5 ) #5 degrees is the default value, though


#
## hbda - distance/angle bb hbond term
##
#protocol.initHBDA('hbda.tbl')
#potList.append( XplorPot('HBDA') )


#New torsion angle database potential
#
from torsionDBPotTools import create_TorsionDBPot
torsionDB = create_TorsionDBPot('torsionDB', system='protein')
potList.append( torsionDB )
rampedParams.append( MultRamp(.002,2,"torsionDB.setScale(VALUE)") )#first differences.


#
# Statistical torsion angle potential
#
from torsionDBPotTools import create_TorsionDBPot
torsionDBPot = create_TorsionDBPot('tDB')
potList.append( torsionDBPot )
```

```
rampedParams.append( MultRamp(.002,2,"torsionDBPot.setScale(VALUE)") )



#covalent terms
from xplorPot import XplorPot
for term in ('BOND', 'ANGL', 'IMPR'):
    potList.append( XplorPot(term) )
    pass

# Set threshold for terms in potList to allow violation analysis.
potList['ANGL'].setThreshold(5.0)   # default is 2.0
potList['IMPR'].setThreshold(5.0)   # default is 2.0
# Use default values for the rest (bond: 0.05, cdih: 5.0, noe: 0.5).

rampedParams.append(MultRamp(0.4, 1.0, "potList['ANGL'].setScale(VALUE)"))
rampedParams.append(MultRamp(0.1, 1.0, "potList['IMPR'].setScale(VALUE)"))

from simulationTools import analyze
print( analyze(potList) )



# Give atoms uniform weights, except for the anisotropy axis
#
protocol.massSetup()
```

```
# IVM setup
#   the IVM is used for performing dynamics and minimization in torsion-angle
#   space, and in Cartesian space.
#

# IVM object used to randomize N-terminal tail
from ivm import IVM
randomizeIVM = IVM()
randomizeIVM.group("resid 36:301 and name C CA N")

protocol.torsionTopology(randomizeIVM)



from ivm import IVM
dyn = IVM()



protocol.torsionTopology(dyn)


# minc used for final cartesian minimization
#
minc = IVM()
protocol.initMinimize(minc)
```

```
protocol.cartesianTopology(minc)
#minc.fix("resid 110:116 and name CA N O C")



# object which performs simulated annealing
#
from simulationTools import AnnealIVM
temp_ini = 3500.0
temp_fin = 25.0

#def accept(potList):
#    """
#    return True if current structure meets acceptance criteria
#    """
#    if potList['noe'].violations()>0:
#        return False
#    if potList['rdc'].rms()>1.2: #this might be tightened some
#        return False
#    if potList['CDIH'].violations()>0:
#        return False
#    if potList['BOND'].violations()>0:
#        return False
#    if potList['ANGL'].violations()>0:
#        return False
#    if potList['IMPR'].violations()>1:
```

```
#       return False
#
#   return True
#
def calcOneStructure(loopInfo):
    """ this function calculates a single structure, performs analysis on the
    structure, and then writes out a pdb file, with remarks.
    """

    # Generate an initial structure by randomizing torsion angles.
    #from monteCarlo import randomizeTorsions
    #randomizeTorsions(randomizeIVM)
    #protocol.fixupCovalentGeom(maxIters=100, useVDW=1)

    #
    # High Temperature Dynamics Stage.
    #
    # Set torsion angles from restraints.
    # (They start satisfied, allowing the shortening of high temp dynamics.)
    #from torsionTools import setTorsionsFromTable
    #setTorsionsFromTable(torsionFile)

    # Initialize parameters for high temp dynamics.
    from simulationTools import InitialParams
    InitialParams(rampedParams) # overides some in rampedParams
    InitialParams(highTempParams) # overides some in rampedParams
```

```
#minimization with all-atom repel potential
protocol.initMinimize(dyn,
                   potList=[term for term in potList
                           if term.instanceName() not in
                           "eefxpot psol noe".split()],
                   numSteps=800 if quick else 800,
                   printInterval=10)



dyn.run()
#minimization with all-atom repel potential
protocol.initMinimize(dyn,
                   potList=[term for term in potList
                           if term.instanceName() not in
                           "eefxpot psol".split()],
                   numSteps=800 if quick else 800,
                   printInterval=10)



dyn.run()

protocol.writePDB("t.pdb")
print( analyze(potList) )
#exit()
```

```
#
# Torsion angle minimization.
#
protocol.initDynamics(dyn,
                      potList=[term for term in potList
                               if term.instanceName() not in
                               "eefxpot psol".split()],
                      bathTemp=temp_ini,
                      initVelocities=1,
                      finalTime=30,  # run for finalTime or
                                     # numSteps * 0.001, whichever is less
                      numSteps=300 if quick else 30000,
                      printInterval=100)

dyn.setETolerance(temp_ini/100) # used to det. stepsize. default: temp/1000
dyn.run()



#minimization with all-atom repel potential
protocol.initMinimize(dyn,
                      potList=[term for term in potList
                               if term.instanceName() not in
                               "eefxpot psol".split()],
                      numSteps=800 if quick else 800,
```

```
                    printInterval=10)



dyn.run()

InitialParams(rampedParams)

#minmization with EEFx potential
protocol.initMinimize(dyn,
                    potList=potList,
                    numSteps=200,
                    printInterval=10)
dyn.run()

#dynamics with EEFx potential
protocol.initDynamics(dyn,
                    potList=[term for term in potList
                            if term.instanceName() not in
                            "eefxpot".split()],
                    bathTemp=temp_ini,
                    initVelocities=1,
                    finalTime=30,  # run for finalTime or
                                  # numSteps * 0.001, whichever is less
                    numSteps=300 if quick else 30000,
                    printInterval=100)
```

```
dyn.setETolerance(temp_ini/100) # used to det. stepsize. default: temp/1000
dyn.run()


#
# Simulated Annealing Stage.
#
# Initialize parameters for annealing.
InitialParams(rampedParams)

# Set up IVM object for annealing.
protocol.initDynamics(dyn,
                potList=[term for term in potList
                      if term.instanceName() not in
                      "eefxpot".split()],
                finalTime=0.4,  # run for finalTime or
                         # numSteps * 0.001, whichever is less
                numSteps=20 if quick else 201,
                printInterval=100)

# Set up cooling loop and run.
from simulationTools import AnnealIVM
AnnealIVM(initTemp=temp_ini,
        finalTemp=temp_fin,
        tempStep=100 if quick else 12.5,
```

```
        ivm=dyn,
        rampedParams=rampedParams).run()

#
# Torsion angle minimization.
#
protocol.initMinimize(dyn,
                potList=[term for term in potList
                        if term.instanceName()!="eefxpot"],
                printInterval=50)
if not quick: dyn.run()

#
# Final Cartesian minimization.
#
protocol.initMinimize(minc,
                potList=[term for term in potList
                        if term.instanceName()!="eefxpot"],
                dEPred=10)
if not quick: minc.run()

#do analysis and write structure when this function returns
protocol.writePDB(loopInfo.filename()+".full",
              selection=AtomSel("all",symSim))
pass
```

```
from simulationTools import FinalParams

from simulationTools import StructureLoop

StructureLoop(numStructures=numberOfStructures,

        doWriteStructures=True,  #analyze and write coords after calc

        averageFitSel=None,

        averagePotList=potList,

        averageContext=FinalParams(rampedParams),

        #calcMissingStructs=True, #calculate only missing structures

        pdbTemplate="SCRIPT_STRUCTURE.sa",

        structLoopAction=calcOneStructure,

        genViolationStats=True,

        averageTopFraction=0.2, #report only on best 20% of structs

#         averageAccept=accept,   #only use structures which pass accept()

        averageFilename="SCRIPT_ave.pdb",    #generate regularized ave structure

        averageCompSel="not resname ANI and not name H*"     ).run()
```
